# A one-base therapeutic insertion in the *HBG2* distal promoter reactivates γ-globin expression

**DOI:** 10.1186/s40164-025-00626-7

**Published:** 2025-03-28

**Authors:** Xiuqin Bao, Yuanyi Gao, Xiaoyi Chen, Zhongju Wang, Xiaoqin Feng, Liren Wang, Jing Du, Yuhua Ye, Feijing Chen, Li Du, Aihua Yin, Xiangmin Xu

**Affiliations:** 1 Medical Genetics Center, Guangdong Women and Children Hospital, Xingnan Road 521, Guangzhou, 510010 Guangdong China; 2 Innovation Center for Diagnostics and Treatment of Thalassemia, Nanfang Hospital, Southern Medical University, Guangzhou, 510515 Guangdong China; 3 Maternal and Children Metabolic-Genetic Key Laboratory, Guangdong Women and Children Hospital, Guangzhou, 510010 Guangdong China; 4 Thalassemia Diagnosis Center, Guangdong Women and Children Hospital, Guangzhou, 510010 Guangdong China; 5 Department of Medical Genetics, School of Basic Medical Sciences, Southern Medical University, Guangzhou, 510515 Guangdong China; 6 Guangzhou Medical University, Guangzhuo, Guangdong China; 7 Department of Pediatrics, Nanfang Hospital, Southern Medical University, Guangzhou, 510515 Guangdong China; 8 Shanghai Key Laboratory of Regulatory Biology, Institute of Biomedical Sciences and School of Life Sciences, East China Normal University, Shanghai, 200241 China; 9 Department of Hematology, Nanfang Hospital, Southern Medical University, Guangzhou, 510515 China

**Keywords:** β-thalassemia, *HBG2* distal promoter, One-base therapeutic insertion, Therapeutic target, Methylation, CD34^+^ HSPC cells, FOXO3

## Abstract

**Background:**

The reactivation of developmental silenced γ-globin genes (*HBG1/2*) has shown promise as a therapeutic strategy for improving symptoms of β-hemoglobinopathies. Currently, the focus of therapeutic targets is primarily on the major fetal hemoglobin suppressors, such as BCL11A and ZBTB7A and of their binding sites on the proximal *HBG* promoter. However, the role of the distal *HBG* promoter in regulating gene expression remains to be explored.

**Methods:**

We used CRISPR/Cas9 system to edit the distal *HBG* promoter. In vitro and in vivo assays, as well as engrafted NCG-Kit-V831M mice, were used for functional validation and mechanistic studies.

**Results:**

We discovered an insertion of nucleotide A (insA) between − 1368 and − 1369 bp upstream of the TSS in *HBG2* resulting in remarkable increase in γ-globin expression in HUDEP-2 cells. We also observed elevated γ-globin expression in human CD34^+^ erythroid progenitor cells from healthy individuals and those with β-thalassemia when introducing insA mutation. Similarly, engrafted NCG-Kit-V831M mice showed increased γ-globin expression. Importantly, neither did insA have any off-target effects nor did it affect the maturation of erythroid cells. Furthermore, we found that the insA mutation created a binding site for the transcription activator FOXO3, which was activated by AMPK. Additionally, introducing insA specifically demethylated the − 162 CpG site on *HBG* promoter by reducing the enrichment of DNA methyltransferase 3 A (*DNMT3A*). At the same time, it activated histone modifications and RNA polymerase II (Pol II) in both distal and proximal *HBG* promoter and might inhibit the binding of BCL11A and ZBTB7A on -115 and − 200 sites on the *HBG* promoter respectively. In addition, combination of insA and the − 115 or -200 editing targets resulted in an amplify effect in reactivating γ-globin genes expression.

**Conclusions:**

Overall, we presented the preclinical data to support the role of insA on regulating γ-globin expression using CD34^+^ HSPC cells derived from healthy donors or patients with β-thalassemia, and subsequently engrafted mice. Our study suggests that introducing insA mutation leads to significantly boosted fetal globin levels and uncovers new safe therapeutic target or strategy for β-hemoglobinopathies.

**Supplementary Information:**

The online version contains supplementary material available at 10.1186/s40164-025-00626-7.

## Background

Reactivating γ-globin or fetal hemoglobin (HbF, α2γ2) expression, which is silenced after birth, has been a promising therapeutic strategy for β-thalassemia and sickle cell disease [1–3]. In recent years, an increasing number of HbF regulatory factors have been discovered [4–11], among which transcription factors (TFs) represented by BCL11A and LRF/ZBTB7A are known to repress the expression of γ-globin through binding to *cis*-acting elements in γ-globin promoter [6, 12]. Additionally, the presence of abnormally high levels of HbF in adult life, known as hereditary persistence of fetal hemoglobin (HPFH) also ameliorate the debilitating clinical manifestation of β-thalassemia and sickle cell disease [5, 8, 11, 13, 14]. HPFH can be caused by large β-globin locus deletions or single point mutations or small deletions within the *HBG* promoter. Single point mutations-HPFH in the proximal *HBG* promoter, particularly in the − 115 and − 200 (relative to the transcription start site (TSS)) cluster [5], disrupt the binding of the major HbF modifiers BCL11A, LRF/ZBTB7A or create a new binding site for the erythroid master regulators KLF1 and GATA1 [5, 8, 14].

The identification of these HbF modifiers and natural occurring mutations has provided potential therapeutic targets for genetic treatment of β-hemoglobinopathies, and some of these are currently being explored. Wu et al. [15] and Frangoul et al. [17] have reactivated the expression of γ-globin by editing the + 58 *BCL11A* erythroid enhancer, resulting in the inhibition of BCL11A and consequently benefiting β-thalassemia and sickle cell disease (SCD) patients by maintaining therapeutic levels of HbF to remarkably reduce their transfusion dependencies. In addition, editing the non-deletional HPFH mutations in *HBG* proximal promoter to activate γ-globin has also been employed as a gene therapy strategy in clinical trials for β-thalassemia and SCD [18, 19]. These researches confirm that reactivating γ-globin expression is a viable strategy for curing β-hemoglobinopathies. However, the current gene editing targets that can be applied for this strategy mainly involves the intronic enhancers of BCL11A and the proximal promoters of *HBG* genes, suggesting that identifying additional targets might be promising in further increasing the therapeutic efficiencies. *HBG* consists of *HBG1* and *HBG2*, both of which have nearly identical nucleotide sequences, including the proximal promoters. Therefore, editing the proximal *HBG* promoter is easy to lead to a large deletion between *HBG1* and *HBG2* [20, 21]. Giving that it is not known whether the intergenic *HBG2-HBG1* region bears critical functional elements, caution should be exercised in the clinical translation of this approach. Even though base editing can avoid this deletion, the editing efficiency remain to be optimized. Hence, there is still room for optimizing gene therapeutic targets for β-thalassemia. A treatment target that meets the criteria of being safe, locating in non-coding region and non-homologous region, devoid of off-target effects, and capable of robustly reactivating γ-globin expression would be more suitable for β-thalassemia. In this context, we aim to explore the *cis*-elements in the distal *HBG* promoter involved in γ-globin regulation.

To determine the role of distal *HBG* promoter in regulating its expression, we utilized CRISPR/Cas9 system to edit and discovered an insertion of the nucleotide A between − 1368 and − 1369 bp upstream of the TSS in *HBG2*. This insertion is referred to as *HBG2*:c.-1421_-1422insA (insA) and leads to a remarkable increase in γ-globin expression in both HUDEP-2 cells and CD34^+^ hematopoietic stem and progenitor cells (HSPCs) from healthy donors and individuals with β-thalassemia. In vivo and in vitro assays were performed to confirm the role of insA in activating γ-globin. We found that insA mutation creates a binding site for the transcription activator FOXO3 and activates chromatin status and demethylates the − 162 CpG site on the *HBG* promoter. This, in turn, results in a decrease in the binding of γ-globin repressors BCL11A and LRF/ZBTB7A to the − 115 and − 200 site. In addition, combination of insA and the − 115 or -200 editing targets resulted in an amplified effect in reactivating γ-globin gene expression. Our research has discovered insA as a novel gene editing target that efficiently stimulates the expression of γ-globin. This target is located within the distal *HBG2* promoter region and exhibits no off-target effects or disruption to other biological functions.

## Methods

### Cell culture

Human CD34^+^ HSPCs from mobilized peripheral blood of deidentified healthy donors and β-thalassemia patients were obtained from Nanfang Hospital, Guangdong, China, following Guangdong Women and Children Hospital institutional review board approval and patient informed consent. The CD34^+^ HSPCs were separated using the Miltenyi CD34^+^ microbead kit (Miltenyi Biotec). CD34^+^ HSPCs were cultured and differentiated as previous described [16]. Briefly, CD34^+^ HSPCs were thawed on day 0 into X-VIVO 15 (Lonza, 04–418Q) supplemented with 100 ng/ml human SCF, 100 ng/ml human thrombopoietin (TPO) and 100 ng/ml recombinant human Flt3-ligand (Flt3-L). HSPCs were electroporated with Cas9 RNP 24 h after thawing and maintained in X-VIVO media with cytokines. 24 h after electroporation, HSPCs were transferred into erythroid differentiation medium (EDM) consisting of IMDM supplemented with 330 µg/ml holo-human transferrin, 10 µg/ml recombinant human insulin, 2 IU/ml heparin, 5% human solvent detergent pooled plasma AB, 3 IU/ml erythropoietin, 1% L-glutamine, and 1% penicillin/streptomycin. During days 0–7 of culture, EDM was further supplemented with 10^− 6^ M hydrocortisone (Sigma), 100 ng/ml human SCF, and 5 ng/ml human IL-3 (R&D) as EDM-1. During days 7–11 of culture, EDM was supplemented with 100 ng/ml human SCF only as EDM-2. During days 11–18 of culture, EDM had no additional supplements as EDM-3.

HUDEP-2 cells were cultured and differentiated as previous described [22, 23]. Briefly, cells were maintained in StemSpan Serum Free Medium (SFEM, StemCell Technologies, Cat. #09650) supplemented with 50 ng/ml human Stem Cell Factor (hSCF, Peprotech, Cat. #300-07), 10 µM dexamethasone (Sigma, Cat. #D4902), 1 µg/ml doxycycline (Sigma, Cat. #D9891), 3 IU/ml erythropoietin (Amgen, Cat. #55513-144-10), 1% penicillin/streptomycin (ThermoFisher, Cat. #15140122). For differentiation, cells were transferred into growth phase medium consisting of EDM, 100 ng/ml SCF and 1 µg/ml doxycycline as EDM-2, and cultured for 4 days. Then transferred the cells into maturation phase medium (EDM was supplemented with 1 µg/ml doxycycline only as EDM-3) and cultured for 3 days. Finally, transferred the cells into EDM for 2–3 days.

### Clonal culture of HUDEP-2 and CD34+ HSPCs

We used flow cytometric to sort single edited HUDEP-2 cells or CD34^+^ HSPCs in 96 well round bottom plates (Nunc). For HUDEP-2 cells, after 7 days of culture, 1/10 of cells were harvested for genotyping. The selected clones were expanded and cultured for further experiments. For CD34^+^ HSPCs, we changed the cells into EDM-2 media 7 days later in 96-well flat bottom plates (Nunc). After 11 days of culture, 1/10 cells were used to genotyping. The remaining cells were changed into EDM-3 for further differentiation. After 18 days of culture, cells were harvested with sufficient material for RNA isolation and RT-qPCR in biological or technical triplicates.

### CRISPR/Cas9 editing

SgRNA was designed and cloned into Lenti-CRISPR vector (Addgene plasmid ID 52961) through restriction site *BsmB*I. Lentivirus transduction was performed as previously described [24]. For CD34^+^ HSPCs, we chemically modified sgRNAs and utilized RNP electroporation as previously described [16]. Briefly, Electroporation was performed using Lonza 4D Nucleofector (V4XP-3032 for 20 µl Nucleocuvette Strips or V4XP-3024 for 100 µl Nucleocuvettes) as the manufacturer’s instructions. The RNP complex was prepared by mix 18 µg Cas9 protein and 0.3 nmol sgRNA for 20 µl Nucleocuvette Strips and incubated 15 min at room temperature before electroporation. 50 K HSPCs resuspended in 20 µl P3 solution were mixed with RNP and transferred to a cuvette for electroporation with program EO-100.

To knock out the insA, we used homologous recombination technique. Briefly, sgRNA, cas9 and a 150 bp ssDNA as donor were electroporated into insA clone HUDEP-2 cells, and the insA KO single clones were sorted by flow cytometry.

SgRNAs and related PCR primers used in this study were listed in supplemental Table 1 in the supplementary information.

### Measurement of indel frequencies and Off-target analysis

Indel frequencies were measured with cells cultured 5 days after electroporation or transduction. Briefly, cells were harvested and lysed with 10 µl lysis buffer consist of 1 µl protease K, 1 µl PCR buffer and 8 µl nuclear free water for 45 min at 55 ℃, followed by 95℃ for 10 min to inactivate protease K. PCR was then performed as previously described. Sanger sequencing was utilized to genotype. Synthego was used to analyze the indel distribution.

We used Cas-OFFinder to predict the off-target sites. Primers (Supplemental Table 1) were designed to amplify the fragment flanking the predicted sites and deep sequencing was performed. We used the website tool Cas-Analyzer to analyze the off-target rate.

### qPCR

RT-qPCR was used to detect the RNA expression level of γ-globin. RNA samples were isolated using TRIzol (ThermoFisher Scientific, cat #15596018). cDNAs were prepared by reverse transcription using Hifair III 1st Strand cDNA synthesis Suppermix (YEASEN, cat #11141ES60). qPCR reaction was prepared with Hieff qPCR SYBR Green (YEASEN, cat #11201ES08) and run on Roche Real time qPCR machine.

### Western blot

Western Blot was performed as standard protocol. The primary antibodies were as follow: anti-HbF (ab137096, Abcam), anti-GAPHD (129-10312, Ray antibody), anti-lamin A/C (ab238303, Abcam), anti-β-Tublin (ab6046, Abcam); anti-phospho-FOXO3 (Ser413) (8174s), anti-phospho-AMPK (Thr172) (2535T), all from Cell Signaling Technology.

### HPLC

HbF and HbA levels were detected by HPLC. Briefly, 1 × 10^7^ cells were harvested and 100 µl HPLC-grade water was added to lysis. Incubated on ice for 10 min and then subjected to 3 freeze-thaw cycle. HbF level was calculated by Variant II (Bio-Rad Laboratories, USA).

Individual globin chain levels were determined by a Shimadzu Prominence instrument with an SPD-10AV diode array detector and an LC-10AT binary pump (Shimadzu, Kyoto, Japan). Vydac 214TP C4 Reversed-Phase columns for polypeptides (214TP54 Column, C4, 300Å, 5 μm, 4.6 mm i.d. ×250 mm) (Hichrom, UK) were used. A 38-60% gradient mixture of 0.1% trifluoroacetic acid in water/acetonitrile was applied at a rate of 1 mL/min.

### The ratio of Gγ/Aγ globin mRNA expression

To quantify the ratio of Gγ/Aγ globin mRNA expression, the *HBG*: c.410G > C polymorphism in exon 3 of *HBG* mRNA was used as a marker and the ratio of Gγ/Aγ globin was determined based on the G (in Gγ mRNA only) and C (in Aγ mRNA only) allele peaks observed on the sequencing chromatographs from the reverse-transcript PCR products obtained using the BioEdit Sequence Alignment Editor. Reverse transcription from total RNA was performed to generate cDNA template using the PrimeScript RT Reagent Kit (Takara, Dalian, China). Reaction was carried out with 2 µg of total RNA, random hexamers and PrimeScript RT Reagent Kit (Takara) for 10 min at 25 °C, 30 min at 48 °C, 5 min at 95 °C, and sanger sequencing was performed. The Gγ mRNA expression was determined by quantification of the G allele of c.410 in the *HBG* gene. The Aγ mRNA expression was determined by quantification of the C allele of c.410 in the *HBG* gene.

### Multiplex ligation-dependent probe amplification (MLPA)

To examine off-target effects of editing due to the duplicated nature of the *HBG1* and *HBG2* genes, we verified the target region cut within the β-globin gene cluster through copy number analysis with SALSA MLPA Probe P102-C1 HBB (MRC-Holland).

### Flow cytometric analysis

To detect the F cells, flow cytometry was performed with FITC-conjugated anti-human HbF (552829, BD pharmingen) antibody. Cells were fixed with 4% paraformaldehyde at RT for 20 min and permeabilized with 0.1% Triton X-100 for 10 min at RT. After washing with PBS, cells were incubated with the FITC-conjugated HbF antibody for 30 min at RT. Cells were washed following incubation and measured with a BD flow cytometry system. Prior to subjecting cells to analysis by BD FACSMelody cytometer, 5 µl of 7-AAD was added to select for live cells.

To determine the erythroid maturation, cells derived from cultures of HUDEP-2 cells and CD34^+^ HSPCs were stained with FITC-conjugated anti-CD235a (eBioscience, 11-9987-82), PE-CY7-conjugated anti-CD71 (eBioscience, 25-0719-42), APC-conjugated anti-CD233 (Miltenyi, 130-117-713), and PE-conjugated anti-CD49d (eBioscience, 12-0499-42) antibodies. FMO was performed to gating. 7-AAD was used to gate live cells. CD235a positive cells were gated to further analyze the expression of surface markers CD233 and CD49d to determine the terminal erythroid differentiation. CD235a and Hoechst33342 were used to determine enucleation rate.

### Engraftment study

All animal experiments were approved by the Guangdong Women and Children Hospital Institutional Animal Care and Use Committee. Non-irradiated NCG-Kit-V831M (T003802) female mice (4–5 weeks of age) were obtained from GemPharmatech in China. Human CD34^+^ HSPCs were obtained from healthy or β-thalassemia donors under the approval of Guangdong Women and Children Hospital Institutional review board and informed consent was obtained. 0.2–0.6 million edited cells (resuspended in 300 µl DPBS) per mice were injected through tail intravenous. Equal numbers of non-edited CD34^+^ HSPCs were injected into NCG-Kit-V831M mice to serve as controls. Flow cytometric analysis was performed to monitor the human xenograft efficiency 16 weeks post engraftment. Serial engraftments were conducted using retro-orbital injection of bone marrow (BM) cells from the primary recipients. For flow cytometric analysis, BM cells were stained with P55-conjugated anti-hCD45 antibody, APC-conjugated anti-mCD45 antibody, BV421-conjugated anti-hCD19 antibody, BV650-conjugated anti-hCD33 antibody, FITC-conjugated anti-hCD235a antibody and BV510 for live/dead staining. Percentage human engraftment was calculated as hCD45^*+*^ cells/ (hCD45^*+*^ cells *+* mCD45^*+*^ cells) *×*100. B cells (CD19^*+*^) and myeloid (CD33^*+*^) lineages were gated on the hCD45^*+*^ population. Human erythroid cells (CD235a^*+*^) were gated on the mCD45^*−*^hCD45^*−*^ population.

CD235a^+^ cells separated from BM cells were used to extract RNA for qPCR analysis of *HBB* and *HBG* expression. DNA extracted from peripheral blood and BM were used to detect the indel frequencies by Sanger Sequencing after PCR.

### Dual luciferase reporter assay and EMSA

Dual luciferase reporter assay was performed as previous described. Briefly, we inserted *HBG* promoter, consist of insA or WT, into pGL4.18 vector to generate insA/WT-pGL4.18 plasmids. The FOXO3 coding region was inserted into pcDNA3.1 vector to generate FOXO3-pcDNA3.1 plasmid. These plasmids were co-transfected into HUDEP-2 cells with 4D-Nucleofector System (Lonza, Switzerland). 24 h after transfection, cells were harvested and the dual luciferase activity was detected using a WallacVictor V 1420 Multilabel Counter (PerkinElmer, San Jose, CA, USA).

The EMSA assay was performed using LightShift EMSA kit (20148X, Thermo Scientific, USA) according to the manufacturer’s protocol. Probes were designed and synthesized with biotin labeling. Nuclear protein was extracted from HUDEP-2 cells using NE-PER nuclear and cytoplasmic Extraction Regents kit (78833, Thermo Scientific, USA).

### Giemsa staining

Giemsa staining was performed using kit (FD7527) from Fdbio science. Differentiated HUDEP-2 and CD34^+^ cells were uniformly smeared on the slide. Buffer A was added and incubated 30 s, Buffer B was immediately added to neutralize for 5 min. The cytospins were measured by an upright microscope (ECLIPSE Ci-S, Nikon) with objective lens 100×.

### Bisulfite sequencing and cloning

Bisulfite modification of genomic DNA was performed with an EpiArtTM DNA Methylation Bisulfite Kit (EM101, Vzyme, China). Bisulfite treated DNA was purified according to the manufacturer’s protocol and eluted to a final volume of 10 µl. We then performed nested-PCR using 2 µl bisulfite treated DNA as template. The nested-PCR products were subjected to Sanger sequencing or cloning into pMD19T vector (D104, TAKARA, Japan). For Sanger sequencing, the methylation level of each CpG sites was calculated on the basis of the C and T allele sequencing chromatography peaks via BioEdit Sequence Alignment Editor V7.0.9.0 (Carlsbad, USA) ^43^. For cloning, about 20 clones were selected and subjected to Sanger sequencing. The methylation level of each sites was calculated as (C allele clones)/ (total clones)%.

### RNA-sequencing

Total RNA was extracted from InsA and WT HUDEP-2. Library were prepared and subjected to sequencing with 150 bp paired-end reads on the MGI DNBSEQ-T7 platform. Sequenced reads were aligned to the UCSC hg19 (human) genome with HISAT2. The expression level of transcript or isoform was calculates using StringTie. Differential expression genes (DEGs) analysis was performed using DESeq2. 1092 significant DEGs were identified according to the follow criteria:|FoldChange|>2 and qValue < 0.05.

### Co-immunoprecipitation and mass spectrometry (MS)

Immunoprecipitation was performed using wild type and insA clone HUDEP-2 cells (3 × 10^7^). Cells were incubated in RIPA lysis buffer at 4℃ for 30 min, centrifuged at 4 ℃ for 10 min and the pellets were discarded. 20 µl of protein A/G plus agarose (sc-2003, Santa Cruz, USA) was added and incubated at 4 ℃ to the collected supernatant with rotation for 1 h to preclear the immunoprecipitation. Anti-FOXO3 ser413 antibody or IgG were added and incubated at 4 ℃ with stirring for 4 h, followed by addition of 20 µl of protein A/G plus agarose and incubated at 4 ℃ with stirring overnight. The beads were centrifuged and washed 4 times with PBS. The pellets were resuspended in 80 µl PBS and subjected to mass spectrometry.

MS was performed as following described. Crush the sample using a glass rod, and add ddH2O, decolorizing solution, and ACN separately. Oscillate for 5 min, and centrifuge to remove the supernatant. Add TCEP (tris(2-carboxyethyl)phosphine) and CAA (chloroacetamide) for reduction and alkylation at 60 ℃ incubation for 30 min. Oscillate with ACN for 5 min, centrifuge to remove the supernatant, and vacuum dry. Add an appropriate amount of trypsin according to the sample volume, and incubate and oscillate overnight at 37 ℃ for enzymatic digestion. The next day, add peptide extraction solution (ACN/formic acid), sonicate for 10 min, centrifuge to collect the supernatant, and vacuum dry. Desalt using a C18 desalting column, vacuum dry, and store at -20 ℃ for subsequent analysis. The mass spectrometry data was acquired using a Q Exactive HF mass spectrometer coupled with an UltiMate 3000 RSLCnano liquid chromatography system. The peptide samples were dissolved in the loading buffer and injected into the system using an autosampler. The separation was performed on an analytical column (75 μm*25 cm, C18, 1.9 μm, 120Å). A gradient was established using two mobile phases (mobile phase A: 0.1% formic acid, 3% DMSO and mobile phase B: 0.1% formic acid, 3% DMSO, 80% ACN). The flow rate of the liquid phase was set at 300 nL/min. The mass spectrometry data was collected in DDA mode, with each scan cycle consisting of one full MS scan (*R* = 60 K, AGC = 3e6, max IT = 25 ms, scan range = 350–1500 m/z) followed by 20 subsequent MS/MS scans (*R* = 15 K, AGC = 1e5, max IT = 50 ms). The HCD collision energy was set at 27. The isolation window for the quadrupole was set at 1.4 Da. The dynamic exclusion time for ion reacquisition was set at 24 s. The mass spectrometry data was searched using MaxQuant (V1.6.6) software with the Andromeda database search algorithm. The reference database used for the search was the Human protein reference database in Uniprot. The identical proteins were used to perform GO analysis.

### Assay for transposase-accessible chromatin with sequencing (ATAC-seq)

ATAC-seq was performed using TransNGS ATAC-Seq Library Prep Kit for Illumina (KP171, Transgen, China) according to the manufacturer’s protocol. We then used TransNGS ATAC-Seq library amplification SuperMix I and TransNGS Tn5 Index Kit for Illumina to prepare the library. The library was purified using MagicPure Size selection DNA Beads (EC401). And then the ATAC-seq library was sequenced with 150 bp paired-end reads on the Illumina novo-seq X plus platform.

All analyzed reads passed quality control with FASTQC and were mapped onto hg19 with bowtie2. After removal of duplicates with picard, the bam data were ranked, and an index was created with samtools. We used MACS2 to generate peak calls and bdgdiff for differential comparison. Using bamCoverage to calculate base depth for visualization.

### Cell apoptosis

Cell apoptosis was analyzed by using Annexin V-FITC/PI Apoptosis Detection Kit (A211, Vazyme, China). Briefly, 1–5 × 10^5^ cells were harvested and then were centrifuged at 1,800 rpm (300 × g) at 4 °C for 5 minutes, discard the supernatant medium. Digest the cells with trypsin without EDTA, centrifuge at 1,800 rpm (300 × g), 4 °C for 5 minutes, discard the supernatant. Wash the cells twice with pre-chilled PBS, centrifuge at 1,800 rpm (300 × g), 4 °C for 5 minutes each time. Add 100 µl of 1 × Binding Buffer and gently mix to form a single-cell suspension. Add 5 µl of Annexin V-FITC and 5 µl of PI Staining Solution, gently mix; incubate in the dark at room temperature (20 ~ 25 °C) for 10 min. Add 400 µl of 1 × Binding Buffer and gently mix. The stained samples should be analyzed within 1 h using flow cytometry.

### Chromatin Immunoprecipitation (ChIP)

ChIP assay was performed using SimpleChIP Enzymatic Chromatin IP kit (9003, Cell Signaling Technology, USA) according to the manufacturer’s protocol. Four million cells were harvested and fixed with 1% formaldehyde for 10 min at 37 °C, and the reaction was terminated by the addition of glycine. 0.5 µl micrococcal nuclease was added and incubated for 20 min at 37℃ to digest DNA. 10 µl of 0.5 M EDTA was used to stop digestion. Sonication was performed with a Covaris S2 instrument (Covaris). 100 µl of digested chromatin is diluted into 400 µl 1X ChIP Buffer prior to the addition of antibodies. 2 µg of antibodies was added and incubated overnight at 4 ℃ on a rotating shaker. Add 30 µl of Protein G Magnetic Beads to the IP reaction and incubate for 2 h at 4 ℃ with rotation. Beads were sequentially washed once with each of the following buffers: low salt buffer, high-salt buffer. Add 150 µl 1×ChIP Elution Buffer to elute chromatin from the antibody/protein G magnetic beads for 30 min at 65 °C with gentle vortexing (1,200 rpm). Complexes were then eluted from the beads by adding 6 µl 5 M NaCl and 2 µl proteinase K, and incubate 2 h at 65 °C. DNA was extracted for further high-throughput sequencing or for qPCR analysis of target genes. ChIP-seq library was prepared and sequenced with 150 bp paired-end reads on the Illumina novo-seq X plus platform.

All analyzed reads passed quality control with FASTQC and were mapped onto hg19 with bowtie2. After removal of duplicates with picard, the bam data were ranked, and an index was created with samtools. We used MACS2 to generate peak calls with input as the control and set a 200-bp bandwidth in the shifting model to denote the peak with *p* < 0.01.

ChIP-qPCR was performed on a Roche real-time qPCR system with SYBR green. The relative enrichment of DNMT3A/FOXO3/H3K27me3/H3K27ac/H3K4me3/BCL11A/ZBTB7A on the *HBG* promoter was determined relative to the enrichment of IgG or input. The primers used in qPCR are listed in supplemental Table 1.

### Analysis of the intergenic region deletion

To determine if the multiplex editing would cause the deletion of the intergenic sequence between insA and − 115 or -200 sites, we performed quantitative real-time PCR with two pairs of primer (indicated by purple and green half arrows respectively in Fig. 7d); one for intergenic region, the other for outside region, which served as control, in WT and multiplex edited HUDEP-2 cells. The signals from the intergenic region (in-test primers, purple) were normalized to corresponding signals from the outside control region (ex-ctrl primers, green). Data were calculated by the 2^− ΔΔCt^ approach and shown as fold of change compared to corresponding WT HUDEP-2 cells.

### Multiplex editing both distal and proximal *HBG2* promoter

We used CRISPR-Cas9 to edited the − 115 and − 200 sites with 3 sgRNA reported in the previous study [5] in insA clone HUDEP-2 cell. The details were the same as the above description. sgRNA used were listed in Supplementary Table S1 in the supplementary information.

### Statistics analysis

We used unpaired two-tailed Student’s t-test using GraphPad Prism for analyses as indicated in figure legends. The figures were generated by using GraphPad Prism and Adobe Illustrator.

## Results

### Introducing InsA was found to play a role in reactivating HbF

To explore the role of *HBG* distal promoter in regulating γ-globin expression, we utilized publicly available data from the ENCODE project, accessed through the UCSC Genome Browser, to identify potential enhancer regions marked by specific histone modifications, including H3K4me1, H3K27Ac, and H3K4me3 and excluding H3K27me3. In addition, we previously found a γ-globin repressor ERF [25], which has a predicted binding motif located at chromosome 11 position 5,277,382 (hg19, 1370 bp upstream of the TSS). We then found that the publicly available data from different lab consistently showed the specific histone modifications in this region (position 5277382), marked by H3K4me1, H3K27Ac, and H3K4me3 and excluding H3K27me3, may function as an enhancer involved in the regulation of γ-globin expression (Supplementary Fig. 1). Therefore, we then utilized CRISPR/Cas9 technology to edit the distal *HBG2* promoter (1370 bp upstream of the TSS) in HUDEP-2 cells. Flow cytometric analysis was performed using HbF antibody to sort the cells expressing HbF and deep sequencing was carried out to determine the genotype (Supplementary Fig. 2a). We found more than half of the read counts were one base insertions, and the remaining half were wild type (Supplementary Fig. 2b). Further investigation revealed that the one base insertion was a nucleotide A inserted between − 1368 bp and − 1369 bp, termed *HBG2*:c.-1421_-1422insA (Supplementary Fig. 2c).

### Introducing InsA elevates γ-globin expression in HUDEP-2 and CD34+ hematopoietic stem and progenitor cells (HSPCs)

**Fig. 1:**
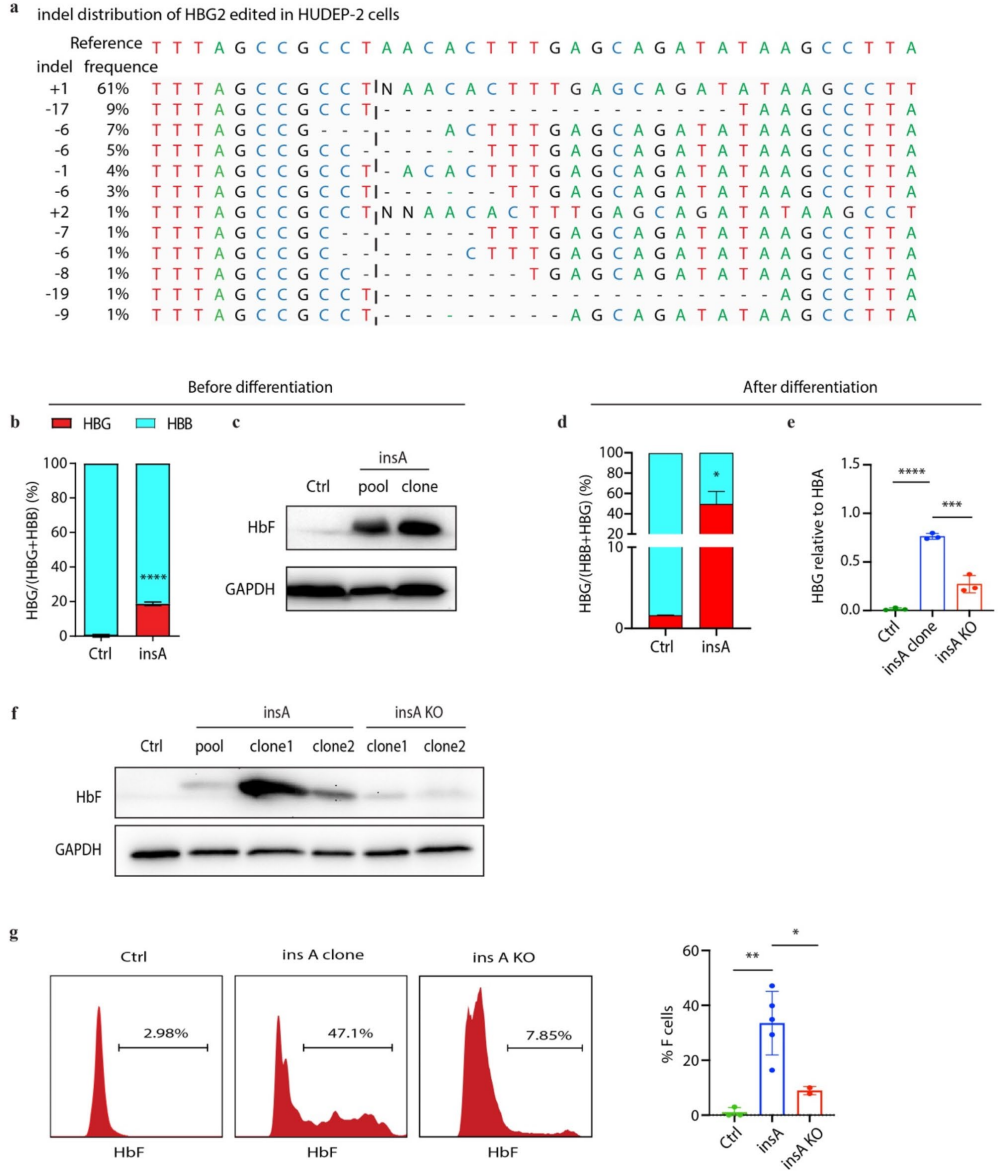
Introducing insA elevates γ-globin expression in HUDEP-2. (**a**) Indel distribution of *HBG2* edited in HUDEP-2 cells analyzed by Synthego webtool after Sanger sequencing. (**b**-**c**) Quantitative real-time PCR and western blot analysis of insA HUDEP-2 cells before differentiation. (**d**-**f**) Quantitative real-time PCR (**d**-**e**) and western blot (**f**) analysis of insA and insA KO HUDEP-2 clones after 8 days differentiation. (**g**) Flow cytometry was performed with anti-HbF antibody to detect the HbF cells in insA and insA KO clones. Ctrl indicated control cells edited in the AAVS1 locus. Data from ≥3 independent experiments are presented as mean ± SD (*p < 0.05, ****p < 0.0001)

To introduce insA in HUDEP-2 cells, we transduced CRISPR/Cas9 and sgRNA with lentivirus. We observed that the indel frequency achieved up to 95%, with the insA mutation accounting for 61% of the total (Fig. 1a and Supplementary Fig. 3a). InsA clones were sorted by flow cytometry. Real-time quantitative polymerase chain reaction (RT-qPCR) revealed a significant increase in the proportion of γ-globin mRNA as a percentage of total β-like globin transcripts, rising from a mean of 0.2–18.8% compared with the control group (Fig. 1b). This result was confirmed by western blot (Fig. 1c). After differentiation, γ-globin level increased from 1.6 to 49.7% (Fig. 1d, e). We further validated the remarkable elevated HbF level using western blot (Fig. 1f) and high-performance liquid Chromatography (HPLC) (Supplementary Fig. 3b), with HbF levels increasing from a mean of 3.5–21.7% in both pool and insA clone HUDEP-2 cells. In addition, the proportion of HbF positive cells (F cells) demonstrated a dramatic increase from 3.0 to 47.1% (Fig. 1g). We then knockout the nucleotide A in insA clone and found that compared with the insA clones, the γ-globin level in insA KO clones were declined measured by qPCR (Fig. 1e), western blot (Fig. 1f) and flow cytometry (Fig. 1g).

**Fig. 2:**
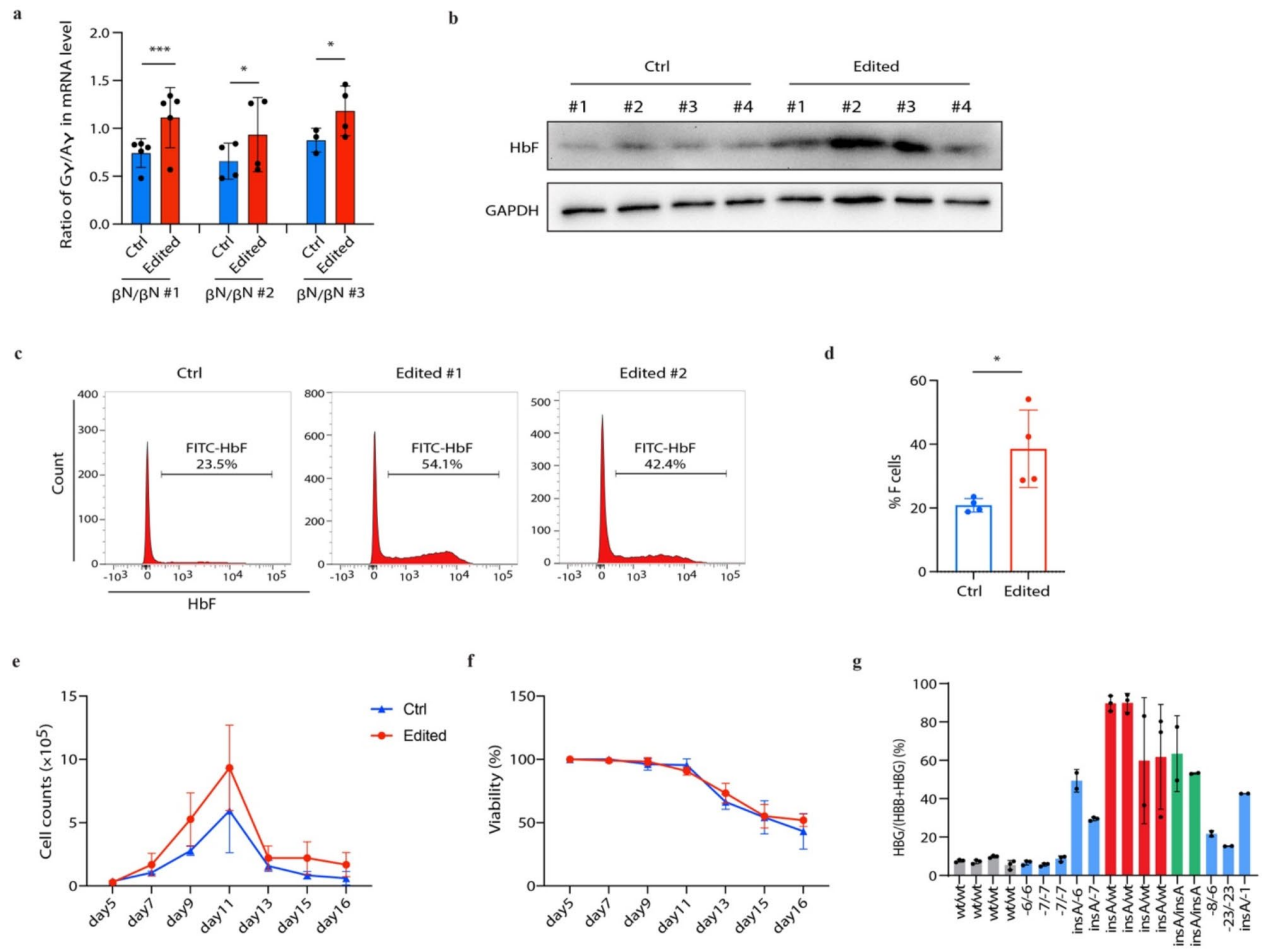
Introducing insA elevates γ-globin expression in CD34^+^ HSPCs. (**a**-**b**) Quantitative measurement of Gγ/Aγ ratio by quantitative real-time PCR (**a**) and HbF production by western blot (**b**) in control (Ctrl) and insA CD34^+^ HSPCs. #1–4 indicated the replicates. (**c**) Flow cytometry was performed to detect the HbF cells in control and insA CD34^+^ HSPCs. (**d**) Statistics result of the HbF cells in control and insA CD34^+^ HSPCs. (**e**-**f**) cell proliferation (**e**) and cell viability (**f**) analysis in control and edited CD34^+^ HSPCs. Ctrl indicated the control cells edited in the AAVS1 locus. (**g**) Quantitative measurement of *HBG* mRNA expression by quantitative real-time PCR in control and insA clone CD34^+^ HSPCs. Data from ≥2 independent experiments are presented as mean ± SD (*p < 0.05, ***p < 0.001, ****p < 0.0001)

To introduce the insA mutation in CD34^+^ HSPCs from healthy individuals, we first optimized the editing efficiency by chemically modifying the sgRNA and optimized the RNP complex concentration. We observed the best RNP concentration, 1:2.75 for Cas9:sgRNA molar ratios (Supplementary Fig. 4a), can result in a 95% indel frequency, with the insA mutation accounting for 60% of the total (Supplemental Fig. 4b, c). We observed a significant increase in the Gγ/Aγ ratio (Fig. 2a). The HbF level measured by western blot (Fig. 2b) and the proportion of F cells measured by flow cytometry were also increased significantly (F cells from 20.85%±2.13–38.58%±12.15%, Fig. 2c, d), compared with the control. Introducing insA did not affect the cell viability and proliferation (Fig. 2e, f). To further validate the role of insA in reactivating γ-globin, we analyzed CD34^+^ edited clone cells and found that, the insA CD34^+^ clones exhibited robustly increased γ-globin expression compared with the wild type clones (Fig. 2g and Supplementary Fig. 5).

### Introducing InsA in β-thalassemia patient HSCs dramatically reactivates γ-globin expression indicating its therapeutic role in β-hemoglobinopathies

**Fig. 3:**
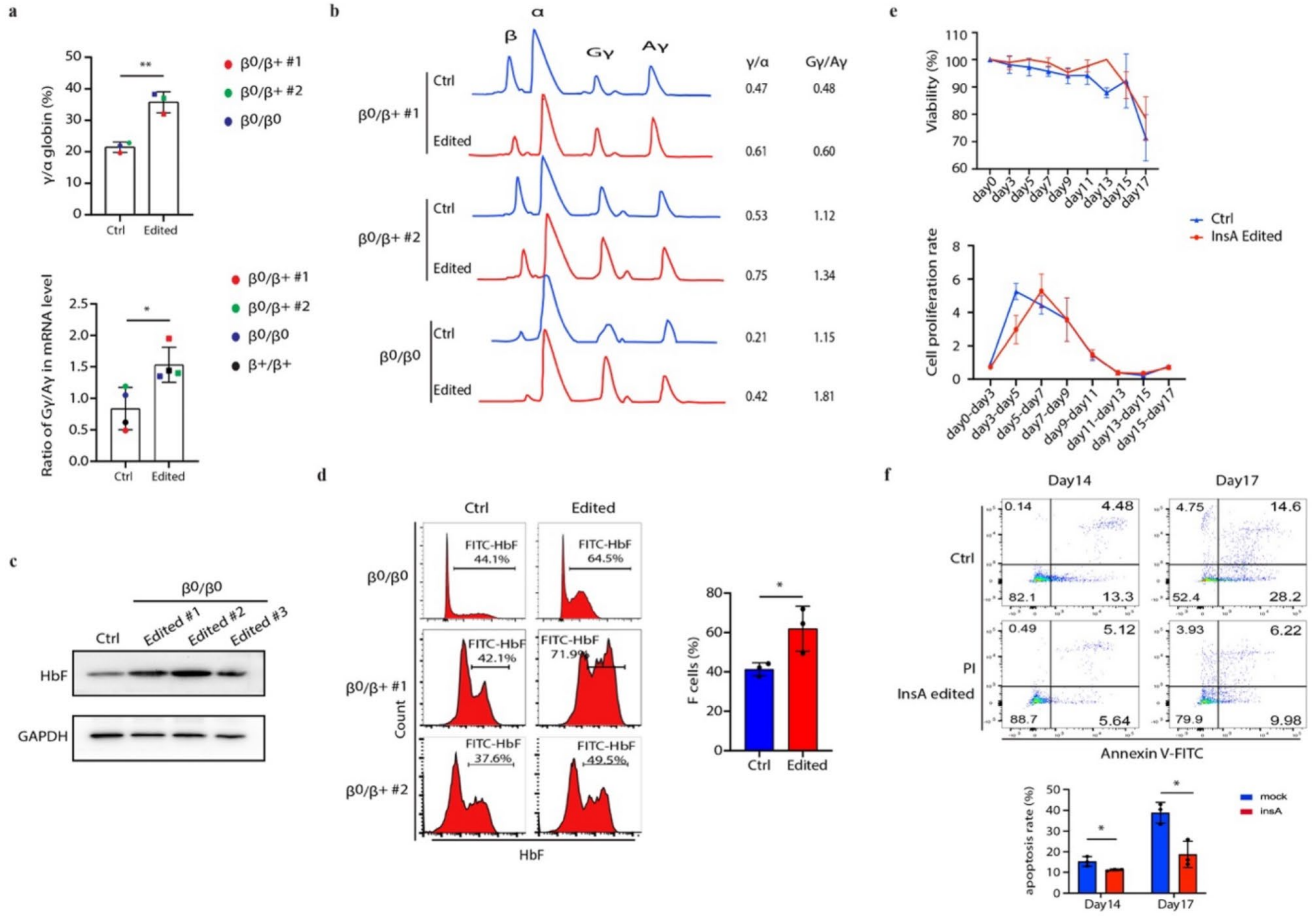
Introducing insA in β-thalassemia patient HSCs dramatically reactivates γ-globin expression. (**a**-**c**) *HBG* mRNA level measured by qPCR (a, top panel) and Gγ/Aγ ratio measured by RT-PCR (a, bottom panel) and protein levels showed as γ/α and Gγ/Aγ by RP-HPLC (**b**) and western blot (**c**) in control and edited CD34^+^ HSPCs derived from β-thalassemia patient. Data are representative of three to four biologically independent replicates. (**d**) The representative image of HbF expression cells measured by flow cytometry (left) and statistics result of HbF cells (right) in control and edited cells. (**e**) Cell viability and cell proliferation rate analysis in control and insA edited cells. (**f**) The representative image of cell apoptosis rate (top panel) and statistics result in control and edited cells (bottom panel)

To test whether introducing insA would result in clinically meaningful γ-globin induction, we edited CD34^+^ HSPCs from four patients with β-thalassemia of varying genotypes, including β^0^/β^0^, β^0^/β^+^ and β^+^/β^+^ (Supplemental Table 2). The RNP editing efficiency was comparable to that observed in healthy donors (ranging from 80 to 96%, Supplementary Fig. 4d). We demonstrated a strong induction of γ-globin, both in mRNA and protein peptide level, in the edited CD34^+^ HSPCs compared to the control cells edited in AAVS1 locus (Fig. 3a, b). The ratio of Gγ/Aγ mRNA level and peptide level were also significantly elevated after introducing insA (Fig. 3a, b). The HbF level detected by western blot (Fig. 3c) showed consistent result with the mRNA level. Additionally, the percentage of F cells in the edited cells increased to 61.97%±11.41% compared 41.27%±3.33% of that in the controls (Fig. 3d). Introducing insA moderate elevated the cell viability and proliferation as observed in the healthy donors (Fig. 3e). The increased γ-globin lead to a significant reduction of cell apoptosis (Fig. 3f).

### InsA reactivates γ-globin expression in transplanted mice

**Fig. 4:**
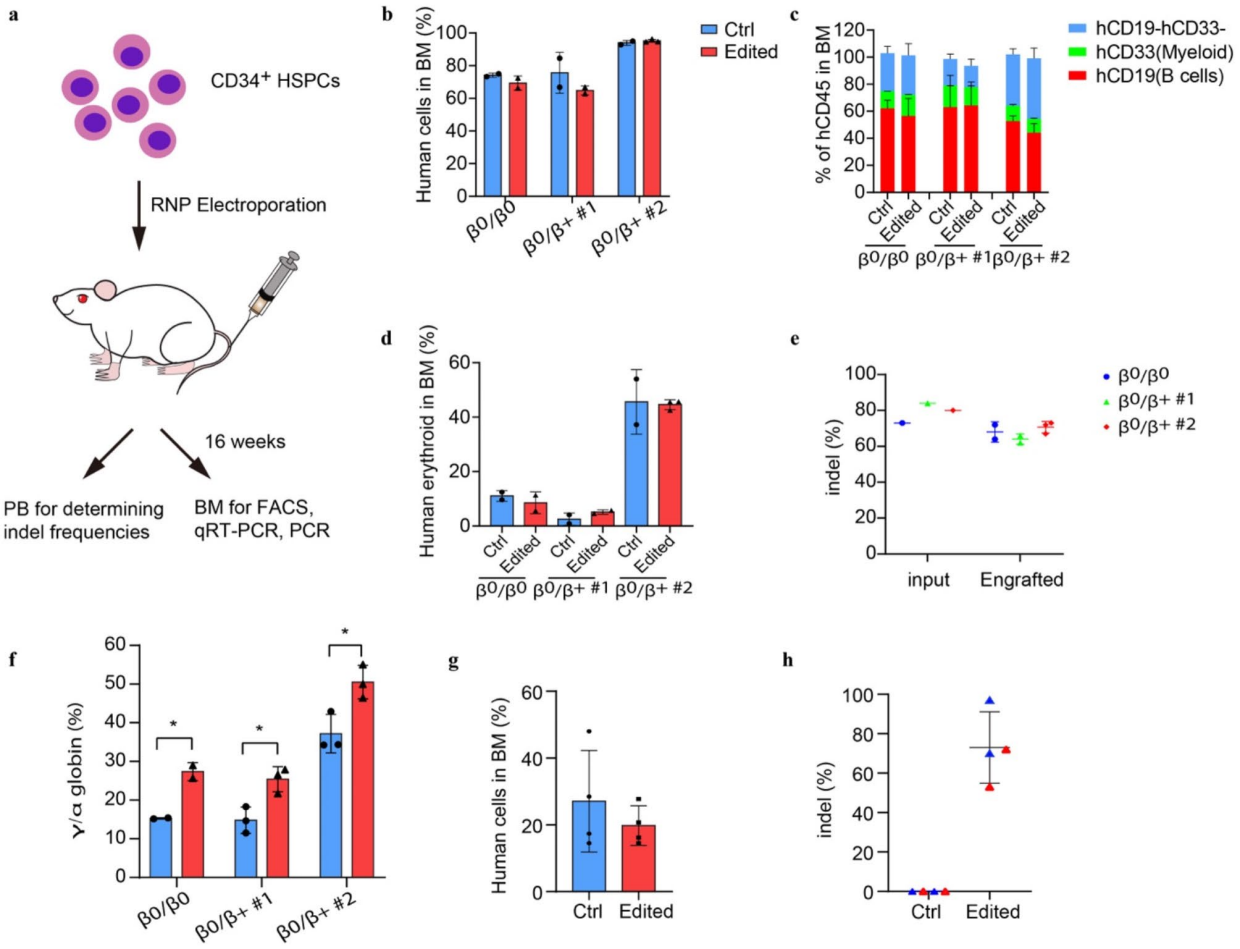
InsA reactivates γ-globin expression in transplanted mice. (**a**) The experimental design for in vivo functional variations of insA. sgRNA and Cas9 protein were electroporated into CD34^+^ HSPCs from three β-thalassemia patient and after 24 h engrafted into immunodeficient mice by intravenous tail injection. Bone marrow (BM) and peripheral blood (PB) were harvested at week 16 after engraftment for further analysis. (**b**-**d**) Flow cytometry analysis in mouse BM 16 weeks after transplantation for determination of human engraftment rates (**b**), human cell multilineage reconstitution (myeloid and B cells, c), and human erythroid cells (**d**). (**e**) Determination of the indel frequencies by Synthego analysis after sequencing of PCR products in BM from engrafted mice. (**f**) Measurement of *HBG* mRNA expression by quantitative real-time PCR in mouse BM after 16 weeks of engraftment. (**g**) BM from two mouse each engrafted with unedited control or edited cells (β^0^/β^+ #2^) were transplanted to four secondary immunodeficient mice. After 16 weeks, BM was analyzed for human cell chimerism by flow cytometry. (**h**) Indel frequencies of insA in BM 16 weeks after secondary transplantation. Data are representative of two to three mice of each group showing as mean ± SD (*p < 0.05, **p < 0.01)

The durability of an autologous hematopoietic cell therapy depends on the ability to permanently modify stem cells. To assess the impact of introducing insA on HSCs, we engrafted edited human CD34^+^ HSPCs from β-thalassemia individuals into immunodeficient NCG-Kit-V831M mice (Fig. 4a, Supplementary Table 3), since they support not only myeloid and lymphoid but also erythroid engraftment. Using three donors from β-thalassemia patients, we observed that the rate of engraftment ranged from 65 to 95% (Fig. 4b), and the recipients of edited and unedited CD34^+^ HSPCs had similar levels of human lymphoid, myeloid, and erythroid cell engraftment within the bone marrow (BM) after 16 weeks (Fig. 4c, d). We further detected the indel frequency in BM from the engrafted mice and noted that, the indel frequencies in edited cells (mean 68%, rang 62-73%, Fig. 4e) were consistent with that of the input cells (Supplementary Fig. 4d). Most importantly, we found significant induction of γ-globin in the edited BM cells (Fig. 4f). Edited BM cells from β^0^/β^+^ donor was able to support the secondary transplantation to a similar level as unedited cells, while maintaining a mean indel frequency of 73% (Fig. 4g, h), consistent with gene editing of self-renewing HSCs. These results provided critical in vivo evidence for the role of introducing insA in activating γ-globin.

### Introducing InsA in *HBG2* distal promoter had little effects on erythroid differentiation and no significant off-target effects

To determine the effect of editing *HBG2* distal promoter on erythroid differentiation, edited HUDEP-2 cells and CD34^+^ HSPCs from healthy or β-thalassemia patients were detected with the surface marker CD235a, CD71, CD233 and CD49d by flow cytometry. Enucleation rate was indicated using Hoechst33342. We observed no differences in the expression of CD235a and CD71 between the edited cells and unedited controls both in HUDEP-2 (Supplementary Fig. 6a) and CD34^+^ HSPCs (Supplementary Fig. 7a, b and 8a). Giemsa staining also confirmed these results (Supplementary Fig. 6b and 7c). However, the rate of late-stage terminal erythroid (CD233^+^/CD49d^low/−^) was slightly increased in edited CD34^+^ HSPCs from β-thalassemia after culture of 18 days (mean 34.7% in edited group compared to 19.95% in unedited control, Supplementary Fig. 8b). Moreover, the enucleation rate significantly increased after introducing insA in CD34^+^ HSPCs from β-thalassemia (Supplementary Fig. 8c, d). These results indicated that introducing insA in *HBG2* distal promoter did not arrest erythroid differentiation in healthy donors but improved terminal erythroid differentiation in β-thalassemia.

To test the specificity of RNP sgRNA in CD34^+^ HSPCs, we conducted deep sequencing to detect the top 13 off-target sites predicted by the Cas-OFFinder. No significant off-target editing events were detected (Supplementary Fig. 9a). Multiplex ligation-dependent probe amplification (MLPA) was also used to confirm the integrity of β-globin cluster (Supplementary Fig. 9b).

### InsA mutation creates a *de novel* site for activator FOXO3

**Fig. 5:**
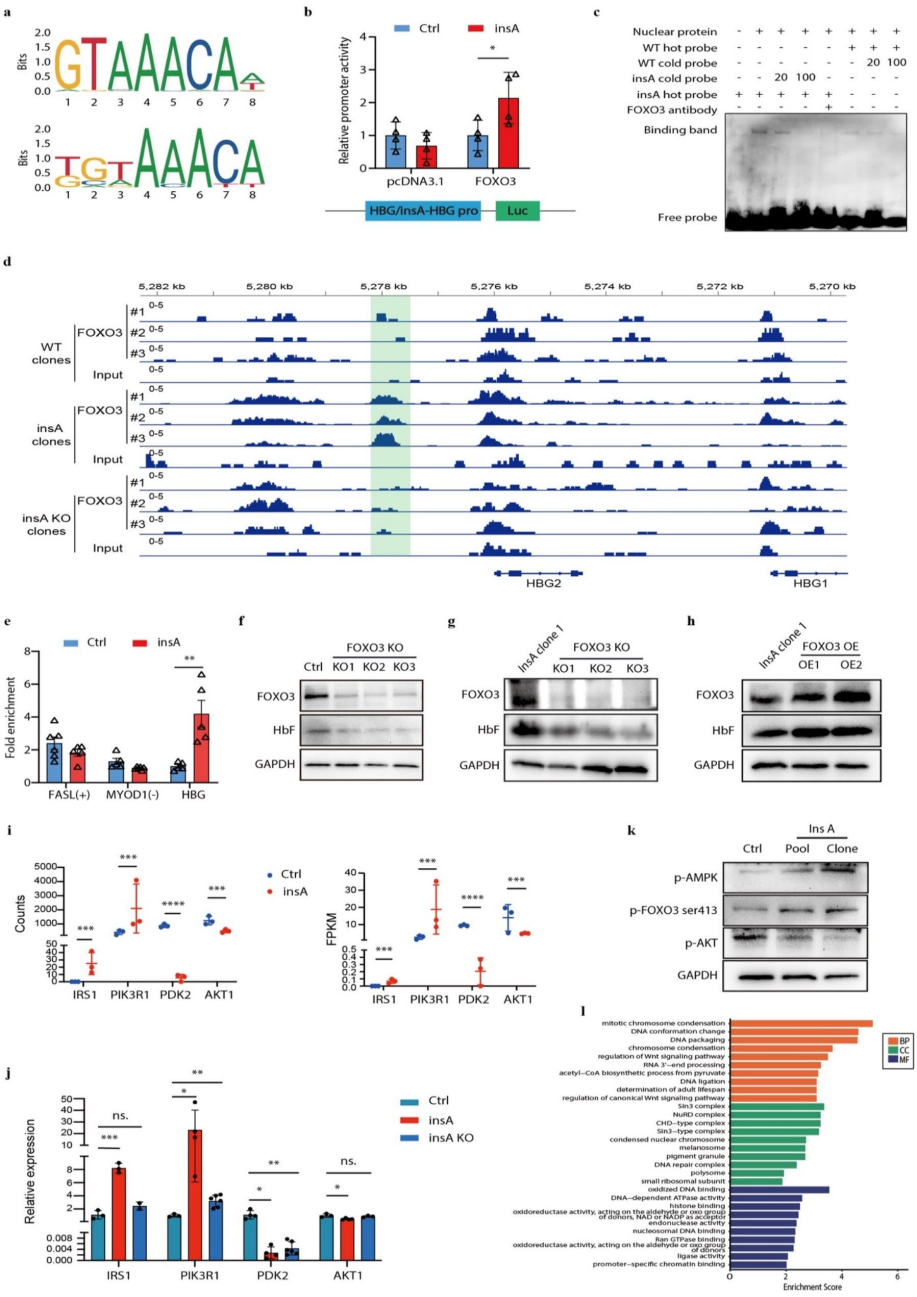
InsA mutation creates a *de novel* site for activator FOXO3. (**a**) The binding motifs of FOXO3 were identified using JASPAR. (**b**) Dual luciferase was performed to determine the interaction between FOXO3 and insA. The wild type (WT) or insA-*HBG* promoter were cloned into the pGL4.18 vector and co-transfected with FOXO3 of pcDNA3.1 into HUDEP-2 cells. (**c**) EMSA was performed with insA or wild type (WT) hot probe, and indicated fold molar excess of the insA or WT cold probe in HUDEP-2 nuclear extract. Anti-FOXO3 antibody was used to perform super shift-EMSA. (**d**) ChIP-seq analysis with anti-FOXO3 antibody in WT, insA and insA KO HUDEP-2 clones. The light green shadow indicated the binding peak of FOXO3 on the HBG2 distal promoter. (**e**) ChIP-qPCR analysis with anti-FOXO3 antibody in control (Ctrl) and insA HUDEP-2 cells. Data from > 3 independent experiments are presented as mean ± SD (**p < 0.01). (**f**-**h**) Western blot analysis in FOXO3 knock out (KO) HUDEP-2 control (**f**) or insA clones (**g**) on day 8 of the differentiation, and in FOXO3 overexpression (OE) in insA HUDEP-2 clone (**h**) without differentiation. (**i**-**j**) The expression level of *IRS1*, *PIK3R1*, *PDK2* and *AKT1* measured by RNA-seq (**i**) and qPCR (**j**) in control, insA and insA KO clones. Data from > 3 independent experiments are presented as mean ± SD (*P < 0.05, **p < 0.01, ***P < 0.001, ns., no significant). (**k**) Western blot analysis with anti-phosphorylated AMPK (p-AMPK), anti-phosphorylated AKT and FOXO3 ser413 antibodies in control (Ctrl) and insA pool or clone HUDEP-2 cells. GAPDH served as control. (**l**) Gene ontology (GO) analysis of the identified proteins pulling down by anti-FOXO3 ser413 antibody in control and insA HUDEP-2 cells. BP, biological process; CC, cellular component; MF, molecular function

To define the mechanism underlying the activation of γ-globin by the insA mutation, we first performed RNA-seq using insA clone HUDEP-2 cells. We found the insA did not affect the expression of the known HbF regulators such as BCL11A, ZBTB7A/LRF, GATA1, KLF1 or MYB, etc. (Supplementary Fig. 10a). We then hypothesized that insA mutation either created or disrupted the binding site for TFs. Using JASPAR, we predicted the binding motif and found 8 TFs that bound to either the wild type or insA mutation (Supplementary Fig. 10b), Among which, we focused on the activator FOXO3 (Fig. 5a), which has been reported to induce HbF [26, 27]. We thus carried out dual luciferase reporter assay to confirm the binding of FOXO3 in insA (Fig. 5b). EMSA, and ChIP-seq performed in insA and insA KO clones, demonstrated the binding of FOXO3 to insA (Fig. 5c-d). ChIP-qPCR performed in insA clones also confirmed this result (Fig. 5e). Knocking out or overexpressing FOXO3 in control and insA clones decreased or increased HbF level respectively (Fig. 5f-h). It was reported that the FOXO3 is activated by AMPK [26, 28–30]. We also found that the genes in AMPK pathway, such as *IRS1* and *PIK3R1*were elevated in insA cells, while *PDK2* and *AKT1* were decreased (Fig. 5i-j). Therefore, we hypothesized that the insA mutation may increase the activation of FOXO3. We conducted a western blot analysis and observed that compared to the unedited control, the activated forms of FOXO3 and AMPK were elevated in insA HUDEP-2 cells (Fig. 5k). We conducted co-immunoprecipitation coupled with mass spectrometry to investigate the potential interactions of the activated form of FOXO3 with other proteins. Gene ontology (GO) analysis revealed that the identified proteins were primarily enriched in histone deacetylase or Sin3-type complexes (Fig. 5l). This indicated that the insA mutation may influence the histone modification pattern of *HBG* promoter.

### InsA activates chromatin status and reduces methylation in *HBG* promoter

**Fig. 6:**
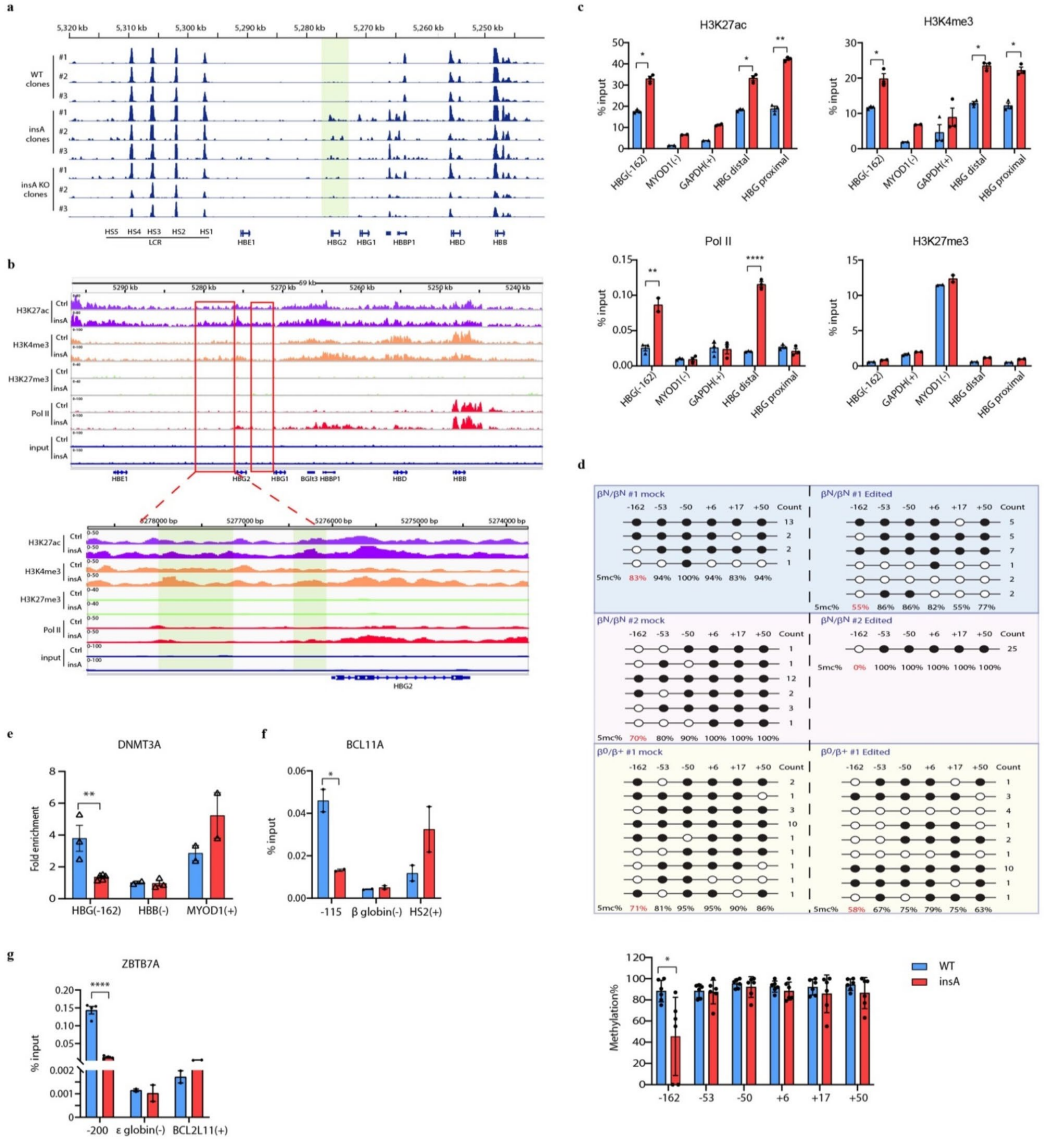
InsA activates chromatin status and reduces methylation in *HBG* promoter. (**a**) ATAC-seq results showed the chromosome accessibility of the β-globin cluster. The light green shadow showed the increased chromosome accessibility on the *HBG2* promoter. (**b**) ChIP-seq binding patterns from control or insA HUDEP-2 cells with anti-H3K27ac, anti-H3K4me3, anti-H3K27me3, and Pol II antibodies at the β-globin cluster. The red box indicated the promoter region of *HBG*. The distal and proximal promoter regions were marked by the light green shadows. (**c**) Detection of the enrichment of the histone modifications by ChIP-quantitative real-time PCR in human CD34^+^ HSPCs. Blue bar, control group; red bar, insA group. -, negative control, +, positive control. (**d**) Top: Analysis of methylation levels at CpG sites (indicated by the distance relative to the transcription start site, TSS) in the *HBG* promoter, evaluated by sequencing. Data were generated with human CD34^+^ HSPCs from individuals with β0-thalassemia. Each row of six CpG sites within a group represents a single bisulfite-treated clone with methylated CpGs (●) or unmethylated CpGs (○). Bottom: the statistical results of methylation level of the two indicated groups. (**e**-**g**) Analysis of the enrichment of DNMT3A on -162 site (**e**), BCL11A (**f**) and ZBTB7A (**g**) on -115 and − 200 site respectively. -, negative control; +, positive control. Data from ≥2 independent experiments are presented as mean ± SD (*p < 0.05, **p < 0.01, ***p < 0.001, ****p < 0.0001)

To further explore the mechanism by which the insA mutation activates γ-globin expression, we performed assay for transposase-accessible chromatin with sequencing (ATAC-seq) in insA and insA KO HUDEP-2 clones to analyze chromatin accessibility, and ChIP-seq to analyze histone modifications, including H3K4 trimethylation (H3K4me3), H3K27 acetylation (H3K27ac), H3K27 trimethylation (H3K27me3) and RNA polymerase II binding (Pol II). We noted that the chromatin accessibility at the *HBG2* locus was increased in insA clones, while it was decreased in insA KO clones (Fig. 6a). The enrichment of active modification marker, H3K4me3 and H3K27ac, were significantly increased both in the *HBG* distal and proximal promoter, while the repressive marker H3K27me3 showed no change compared to the control (Fig. 6b). ChIP-qPCR analysis in CD34^+^ HSPCs also confirmed these results (Fig. 6c). The Pol II was significantly enriched in *HBG* proximal promoter both in ChIP-seq and qPCR results, but the significantly elevated in distal promoter was only observed in Chip-qPCR result (Fig. 6b-c). Histone modifications are often associated with DNA methylation to regulate gene expression [31]. Thus, we examined the methylation levels of the *HBG* promoter and found no differences at other positions, except for a significant decrease at the − 162 CpG site in both HUDEP-2 (Supplemental Fig. 11) and CD34^+^ HSPCs (Fig. 6d, Supplemental Fig. 12). We also observed a significant decrease in the enrichment of DNMT3A at the − 162 CpG site (Fig. 6e). Furthermore, the enrichment of BCL11A and ZBTB7A at the − 115 and − 200 sites, respectively, was significantly reduced (Fig. 6f-g). These findings suggest that the insA mutation activates histone modification in the *HBG* proximal promoter and induces demethylation of the − 162 CpG site, thereby might inhibit the binding of transcription factors BCL11A and ZBTB7A to reactivate γ-globin expression.

### Multiplex mutagenesis in HUDEP-2 cells amplified the induction of HbF level

**Fig. 7:**
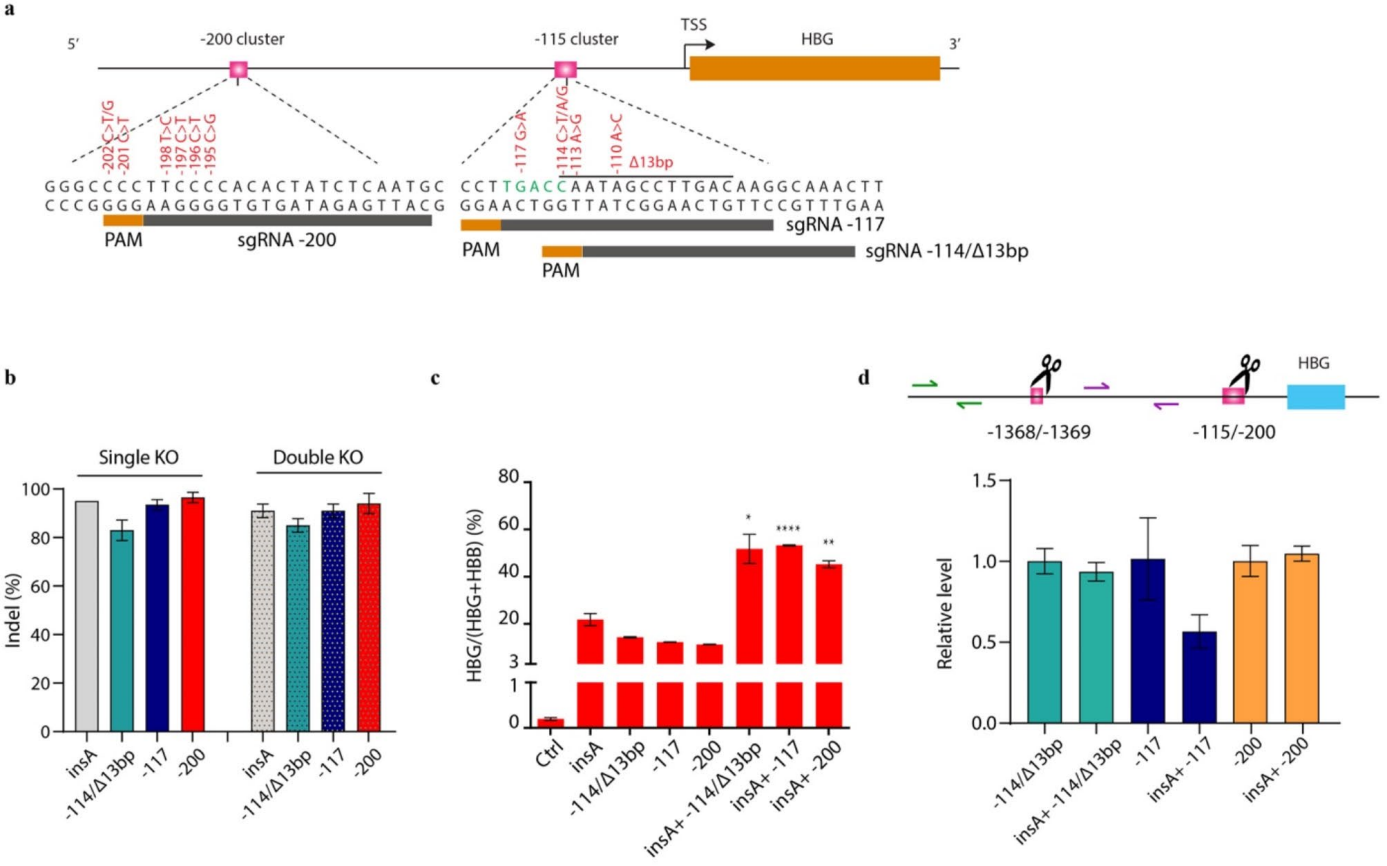
Multiplex mutagenesis in HUDEP-2 cells amplified the induction of HbF level. (**a**) Four modified synthetic (MS) sgRNAs respectively targeting − 115 and − 200 on the *HBG* promoter marked with dark grey boxes. (**b**) The editing rate of the three sgRNAs and insA sgRNA in HUDEP-2 cells. (**c**) *HBG* expression analysis by quantitative real-time PCR in single or multiplex editing in HUDEP-2 cells. Data from 2 biological replicates were showed as mean ± SD (*p < 0.05, **p < 0.01, ***p < 0.001, ****p < 0.0001). (**d**) Top: diagram showing the *HBG* region. The CRISPR-Cas9 cleavage sites are indicated by two scissors. qPCR primers targeting the intergenic sequence between the cleavage sites are indicated (purple half arrows). Another pair of primers binding outside of the deletion region are marked by green half arrows and was used as an internal control to adjust for differences in template DNA quality. Bottom: qPCR was performed to detect the deletion of the intergenic sequences. The signals from the intergenic region (purple primers) were normalized to corresponding signals from the outside control region (green primers). Data were calculated by the 2^− ΔΔCt^ approach and shown as fold of change compared to corresponding wild type HUDEP-2 cells

As a proof of principle, we also assessed whether the effect of insA on HbF can be amplified with editing proximal HPFH natural mutation site. We performed multiplex mutagenesis by editing both insA and the proximal HPFH natural mutation site, which included the − 115 cluster (-114, -117 and Δ13bp) and − 200 cluster (-195, -196, -197, -201 and − 202). Three guide RNAs (sgRNAs) were designed to target these two clusters (Fig. 7a-b, Supplemental Table 1). As expected, all conditions alone elevated the γ-globin mRNA level (Fig. 7c). Interestingly, a cooperative effect was observed when combining the insA mutation with the proximal HPFH mutation site (Fig. 7c). Importantly, this multiplex mutagenesis, except for the sgRNA-117, did not cause any deletions between the insA and the proximal HPFH mutation site (Fig. 7d). The increase in γ-globin caused by the − 117 and insA double editing is likely primarily due to the deletion of the intermediate region, rather than solely the effect of the double editing.

## Discussion

Currently, the most significant modifiers of β-thalassemia are HbF level [32–34]. The *cis*-elements that regulate HbF are primarily focused on the *HBG* proximal promoter, specifically the − 115 and − 200 cluster. In 2006, Higgs et al. found a gain-of-function regulatory SNP (rSNP) in a non-genic region between the α-globin genes and their upstream regulatory elements that creates a potential GATA1 binding site resulting in a new promoter like element that interferes with normal activation of all downstream α-like globin genes [35]. This finding suggests that even in some upstream distal regions that were previously believed to have no important functions, once mutations occur, they can still regulate downstream gene expression. In this study, we discovered that inserting a nucleotide A between positions − 1368 and − 1369 can greatly increase the expression level of γ-globin. The distal promoter sequence does not exhibit homology, so if there is a strong activating effect on the γ-globin, it might be more suitable as a gene therapy target for beta-thalassemia [20, 21]. We presented the preclinical data to support the role of insA on regulating γ-globin expression using CD34^+^ HSPC cells derived from healthy donors (Fig. 2) or patients with β-thalassemia (Fig. 3), and subsequently engrafted mice (Fig. 4). Furthermore, our functional studies demonstrated that insA activated the histone modifications and RNA polymerase II (Pol II) in both distal and proximal *HBG* promoter, and specifically demethylated the − 162 CG site. Observations, made in 2021 [36], that some non-coding sites that do not contain any epigenetic marks can significantly affect cell growth and survival, and are crucial for stabilizing the global chromatin structure also supported our finding.

During the detection of the influence of insA on the methylation level of the *HBG* promoter, we observed a significant decrease in the methylation specifically at position − 162. It is well known that the binding sites of the two major HbF modifiers, BCL11A and ZBTB7A, are respectively located on the − 115 and − 200 cluster [12], which flank the − 162 position. Based on this knowledge, we supposed that the demethylation of the − 162 site mediated by insA may affect the binding of BCL11A and ZBTB7A to the − 115 and − 200 cluster, since reduced DNA methylation typically leads to increased accessibility for activated TFs and decreased accessibility for repressors in the promoter state [5]. Supporting our hypothesis, the results of ChIP-qPCR analysis of histone modification showed elevated enrichment of H3K4me3 and H3K27ac. In addition, RNA-seq analysis of insA HUDEP-2 cells demonstrated no change in the expression of BCL11A and ZBTB7A (Supplementary Fig. 10a), which was in line with erythroid differentiation. This indicated that the reactivation of γ-globin expression mediated by the insA mutations independent of previously identified erythroid repressors and may involve an upstream regulatory element. Considering that allele-specific methylation (ASM) can influence the regulatory ability of a regulatory SNP [37], we proposed that insA may reactivate the γ-globin by demethylating the proximal promoter to inhibit the binding of BCL11A and ZBTB7A to the − 115 and − 200 sites on *HBG* proximal promoter. We observed in our study that the methylation level in *HBG* promoter in -115 and − 200 cluster edited cells were decreased not only at -162 site, but also at other CpG sites, when compared with the control cells. The reduced enrichment of BCL11A and ZBTB7A on the − 115 and − 200 sites on *HBG* proximal promoter primary also supported our hypothesis. However, further experiments, such as electrophoretic mobility shift assay (EMSA), should be carried out to determine the effects of insA on the binding of BCL11A and ZBTB7A to the *HBG* promoter.

The insA mutation was found to create a binding site for FOXO3. However, a limitation of this study is that the underlying mechanism remains to be uncovered. RNA-seq analysis showed no observable change in FOXO3 expression and its activator SIRT1 [38], suggesting that insA did not alter FOXO3 expression but may increase its activated form (p-FOXO3 Ser413) [26, 27]. The RNA sequencing data show that there are many differentially expressed genes between insA and the control group, but the genes that regulate HbF have not changed. These significantly altered genes are mainly enriched in some metabolic pathways and signal transduction pathways (data not showed). This suggests that insA may affect the homeostasis of the intracellular environment, thereby leading to changes in gene expression in some stress pathways. Therefore, we speculate that insA may affect the intracellular environment, thereby influencing the expression of upstream factors in the AMPK pathway, such as *IRS1*, *PIK3R1*, *PDK2* and *AKT1* showed in Fig. 5, which in turn affects AMPK and AKT, leading to the phosphorylation and activation of FOXO3. Site-specific mutation experiments characterizing phosphorylation on FOXO3 should be performed in further study to determine whether insA can activate FOXO3 and thus regulate the expression of γ-globin.

In addition, based on our findings from mass spectrometry analysis, the differences in FOXO3 interacting proteins between the insA and control groups are mainly enriched in the chromatin modification pathway, including HDAC1. Our subsequent research will continue to explore the relationship between FOXO3 and chromatin modification, such as its relationship with HDAC1and the NuRD complex, which have been reported to interact with BCL11A and ZBTB7A to inhibit γ-globin expression. Additionally, we are also conducting research on site-specific methylation modifications, combining insA and site-specific methylation modifications to explore whether it can amplify the activation and expression of γ-globin.

Multiplex genome editing in HSCs is highly efficient while its therapeutic safety has been evaluated [39]. Combining *cis* and *trans* fetal globin reactivation mutations has the potential to significantly increase HbF both totally and on a per cell basis over single editing [39]. In this study, we combined two *cis* fetal globin reactivation mutations, the distal insA and the proximal HPFH point mutation, to enhance the effect of increasing HbF. We first illustrated that multiplex editing of different regulated elements in *HBG* promoter can amplify the effect of insA on HbF reactivation. In thalassemia, higher hemoglobin levels are associated with better outcomes, and patients with transfusion-dependent β-thalassemia (TDT) who maintain pretransfusion hemoglobin levels > 10.5 g/dL have a higher survival rate [40, 41]. Therefore, multiplex editing of insA and the HPFH mutation sites may hold promise as a therapeutic strategy for treating β-thalassemia, particularly in cases requiring higher hemoglobin levels. Future studies should focus on optimizing the indel frequency and minimizing off-target effects in multiplex editing.

Altogether, our study provides a promising target for genetic therapies of β-hemoglobinopathies, since (1) InsA mutation is located on the distal *HBG* promoter and has no off-target effects; (2) insA mutation significantly elevated the expression of γ-globin in CD34^+^ HSPCs from healthy donors or patients with β-thalassemia, as well as in engrafted mice; (3) the editing efficiency of insA can reach up to 95% and maintains this high levels of efficiency even after serials engraftment of BM; (4) introducing the insA mutation does not influence erythroid differentiation or other known erythroid modifiers. (5) introducing insA mutation activates the chromatin state. Moreover, in light of our results, combining insA with demethylation of -162 CpG site or editing proximal HPFH natural mutation site may further improve the therapeutic index.

## Electronic supplementary material

Below is the link to the electronic supplementary material.

Supplementary Material 1

## Data Availability

The data that support the finding of this study are available within the paper, figures, tables, and the supplementary information. The RNA-seq, ChIP-seq and ATAC-seq data are publicly accessible from the National Center for Biotechnology Information GEO database with the accession number GSE262795, GSE262797 and GSE283117 respectively.

## Abbreviations

ATAC-seq: Assay for transposase-accessible chromatin with sequencing
TDT: Transfusion-dependent β-thalassemia
MLPA: Multiplex ligation-dependent probe amplification
EMSA: Electrophoretic mobility shift assay
ASM: Allele-specific methylation
rSNP: Regulatory single nucleotide polymorphism
GO: Gene ontology
HSPCs: Hematopoietic stem and progenitor cells
HPLC: High-performance liquid Chromatography
HPFH: Hereditary persistence of fetal hemoglobin
RT: Room temperature
TSS: Transcription start site
EDM: Erythroid differentiation medium
TPO: Thrombopoietin
SCD: Sickle cell disease
TFs: Transcription factors
BM: Bone marrow
RT-qPCR: Real time quantity polymerase chain reaction
FITC: Fluorescein Isothiocyanate
PE: P-phycoerythrin
APC: Allophycocyanin
MS: Mass spectrometry
H3K4me3: H3K4 trimethylation
H3K27ac: H3K27 acetylation
H3K27me3: H3K27 trimethylation
Pol II: RNA polymerase II binding
