# Supplementary material for: A one-base therapeutic insertion in the HBG2 distal promoter reactivates γ-globin expression
Source: Exp Hematol Oncol. 2025 Mar 28;14:47. doi: 10.1186/s40164-025-00626-7 (PMC11951516; doi:10.1186/s40164-025-00626-7)
Supplement: Supplementary file 1 — Supplementary Material 1 [file 40164_2025_626_MOESM1_ESM.docx]

**Supplemental data**

**
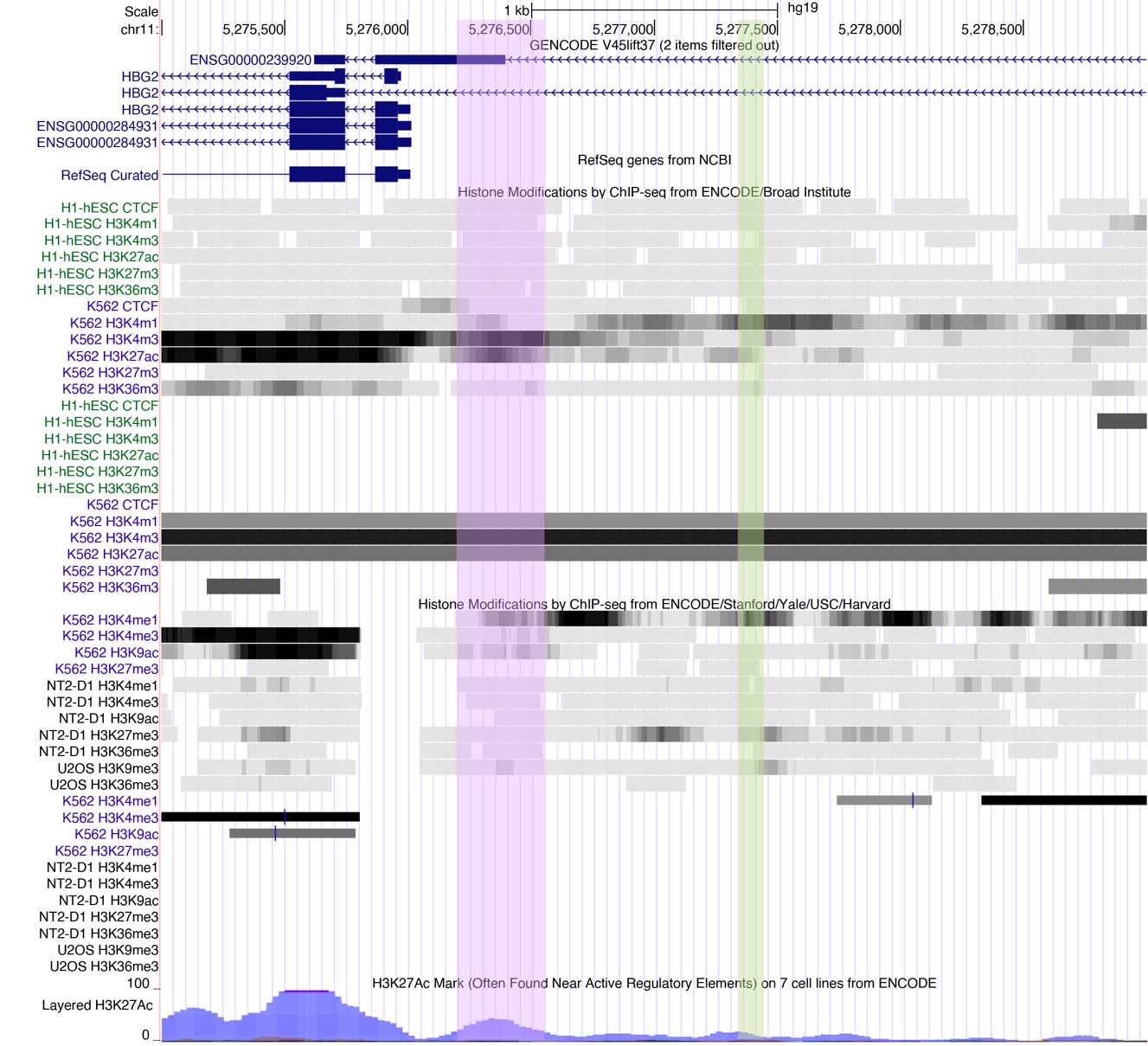
Supplementary Figure 1. The ChIP-seq data attained from ENCODE project showed the histone modification markers on the *HBG2* promoter.** The light purple shadow indicated the region of *HBG2* proximal promoter, while the light green shadow indicated the region of the target on this study, which was marked by increased enrichment of H3K4m1\H3K4m3\H3K27ac, and decreased enrichment of H3K27me.

**
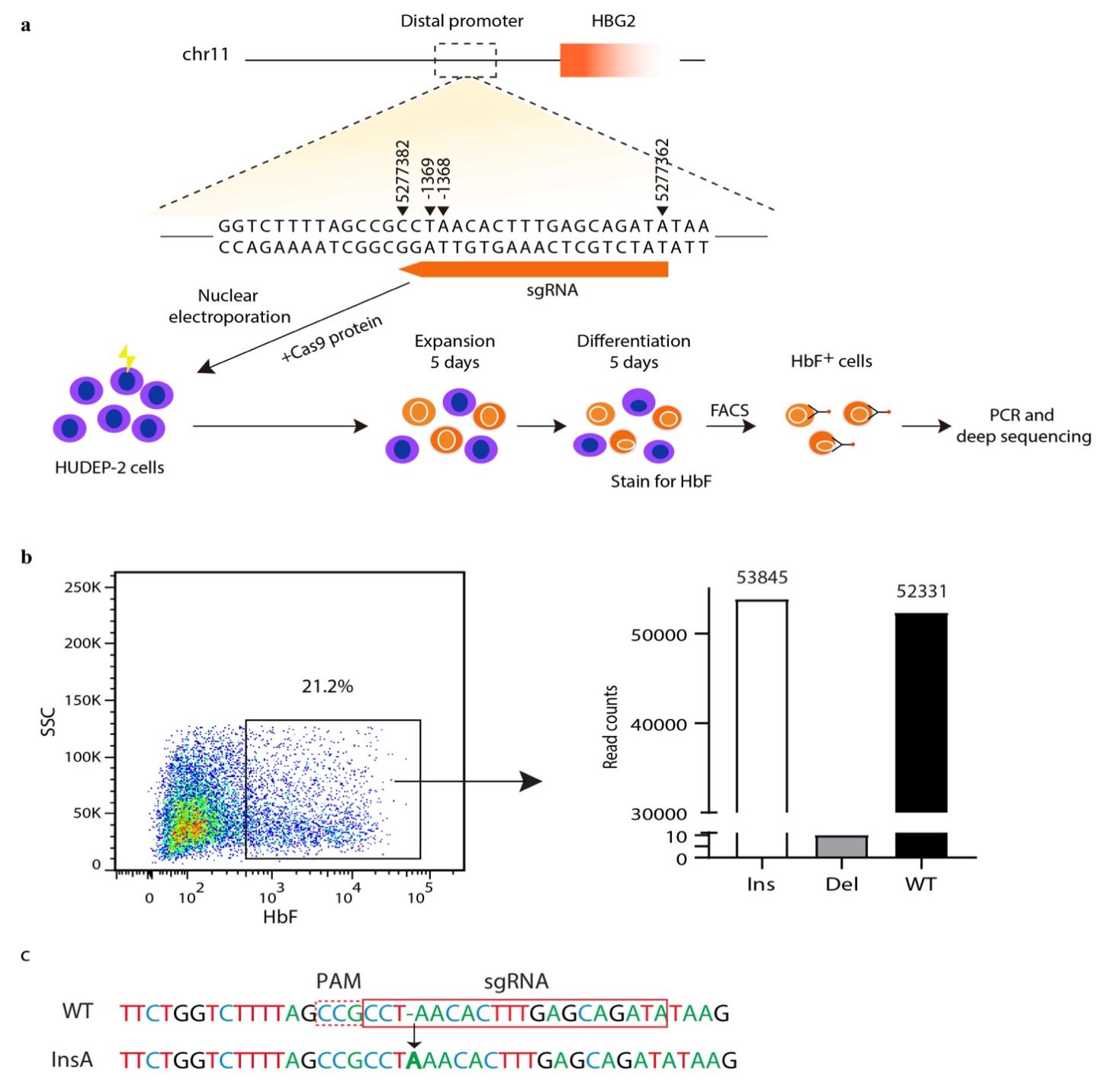
**

**Supplementary Figure 2. Introducing insA was found to play a role in regulating HbF.** a. Screening flow chart of HbF cells. b. Left: flow cytometry of edited HUDEP-2 pools with HbF antibody; right: the distribution of indels in HbF positive cells. c. The position of insA.

**
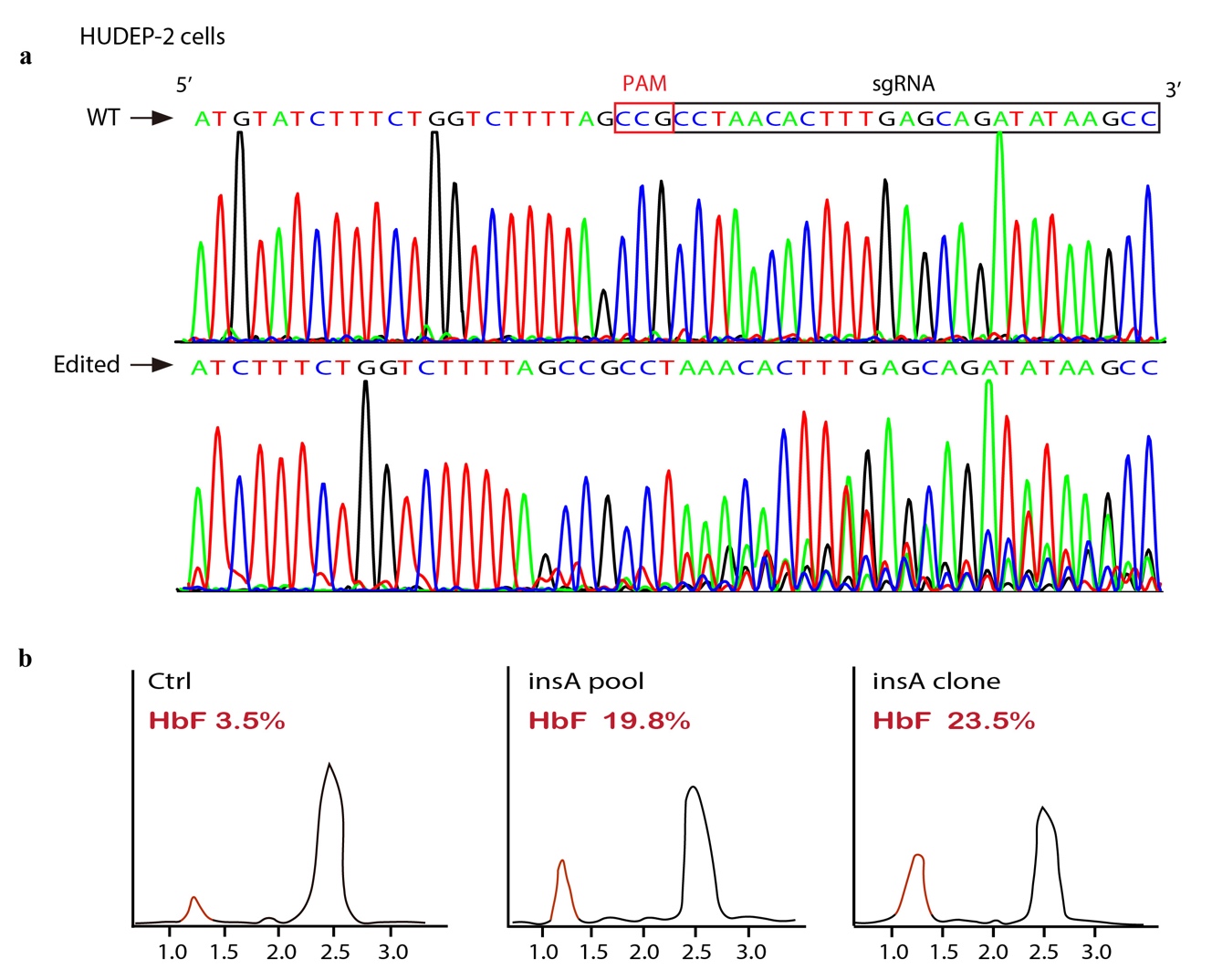
Supplementary Figure 3. Representative sequencing chromatograph of PCR products of edited and WT HUDEP-2 cells. a.** The black box indicates the sgRNA sequence, while the red box denotes the protospacer adjacent motif (PAM). b. Quantitative measurement of HbF in insA HUDEP-2 cells by HPLC.

**
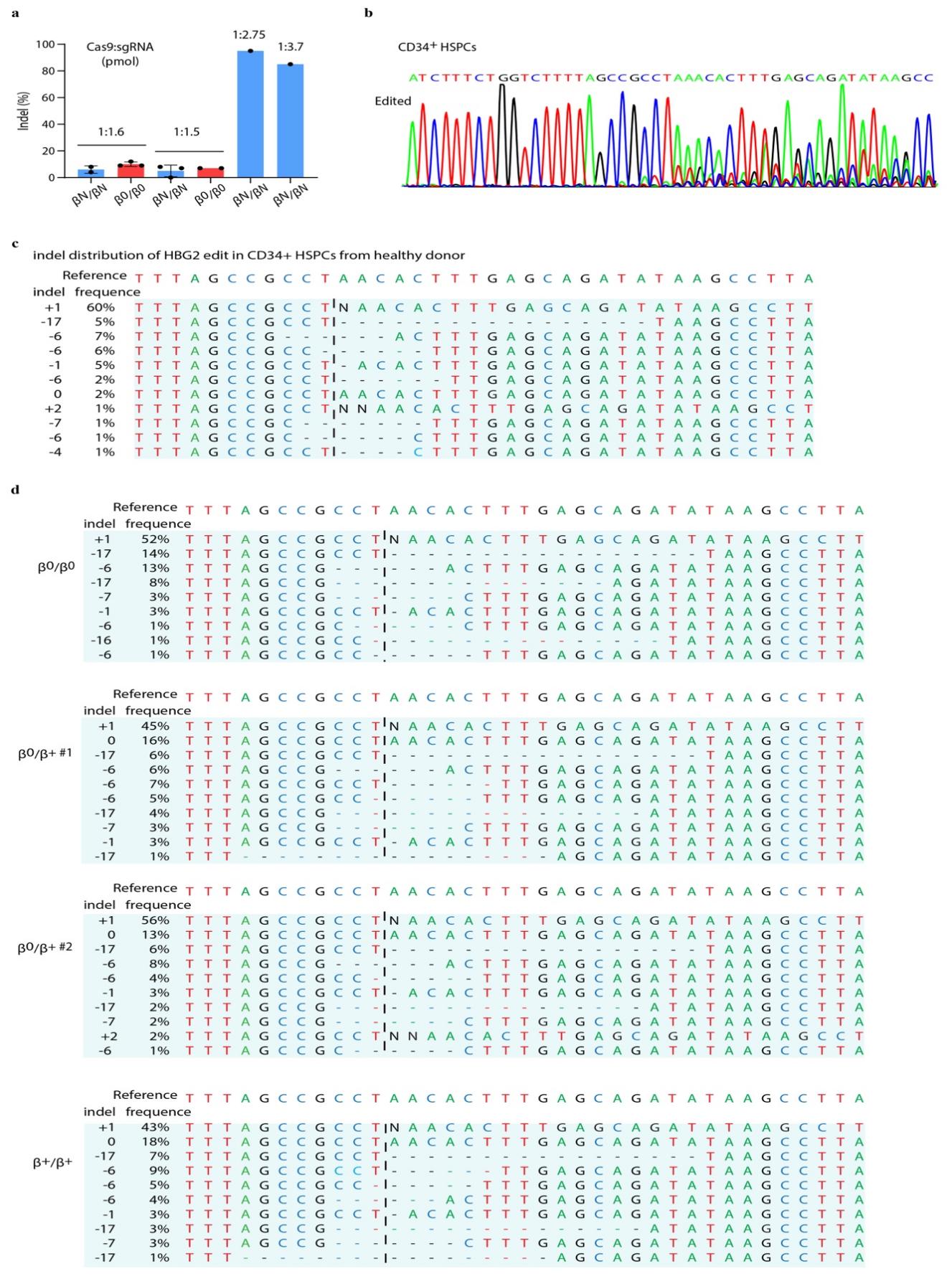
Supplementary Figure 4. Introducing insA in human CD34^+^ HSPCs with CRISPR-Cas9 has a high editing rate.** a. Optimization of the editing rate through different molar ratios of sgRNA and cas9 protein in human CD34^+^ HSPCs from healthy donors and β-thalassemia patients. b. Representative sequencing chromatograph of PCR products of edited CD34^+^ HSPCs cells. c-d. The indel distribution pattern of insA CD34^+^ HSPCs from healthy (c) and β-thalassemia patients (d).

**
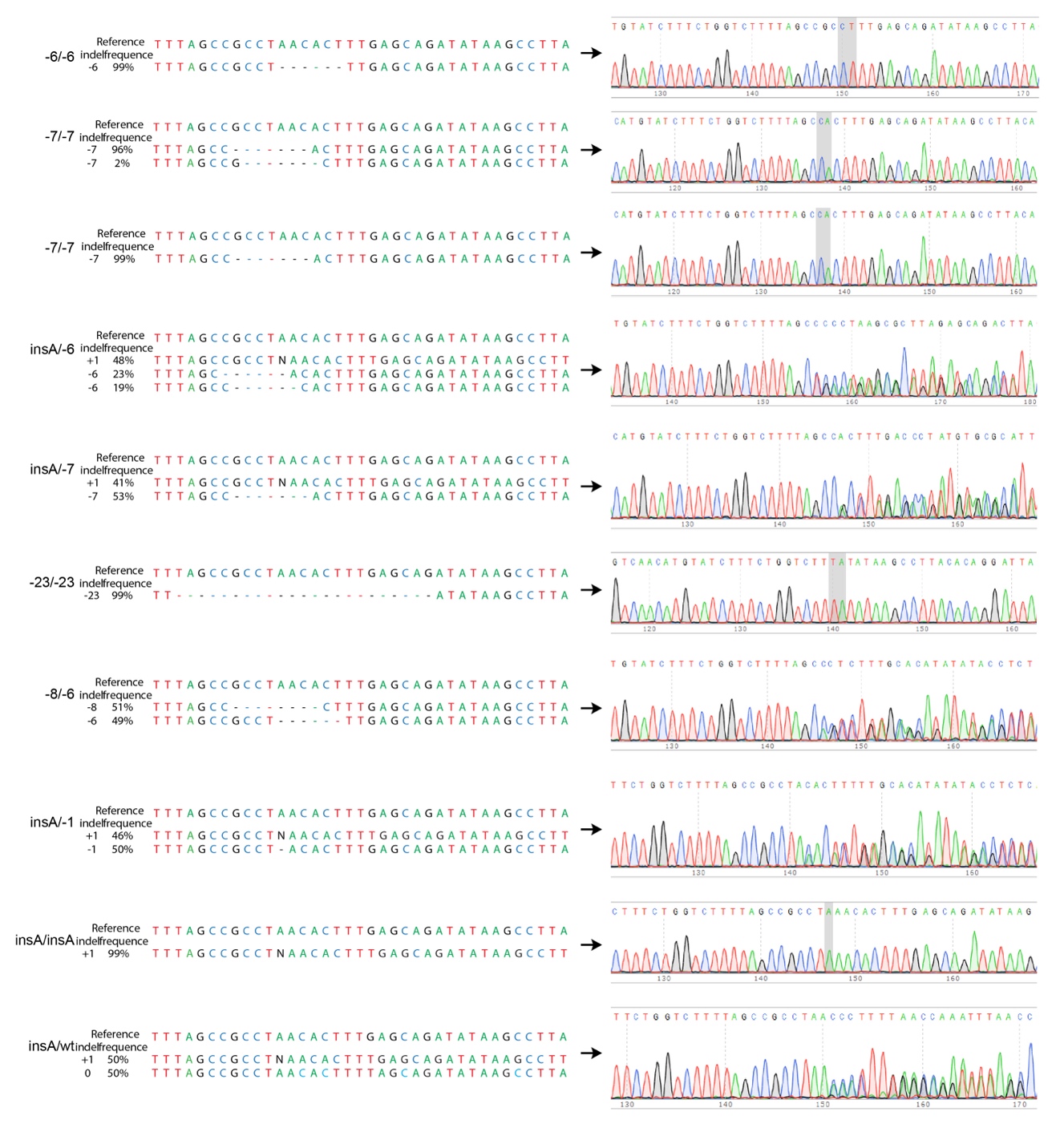
**

**Supplementary Figure 5. The indel pattern and sequencing chromatographs of PCR products of insA edited CD34^+^ clones.**

**
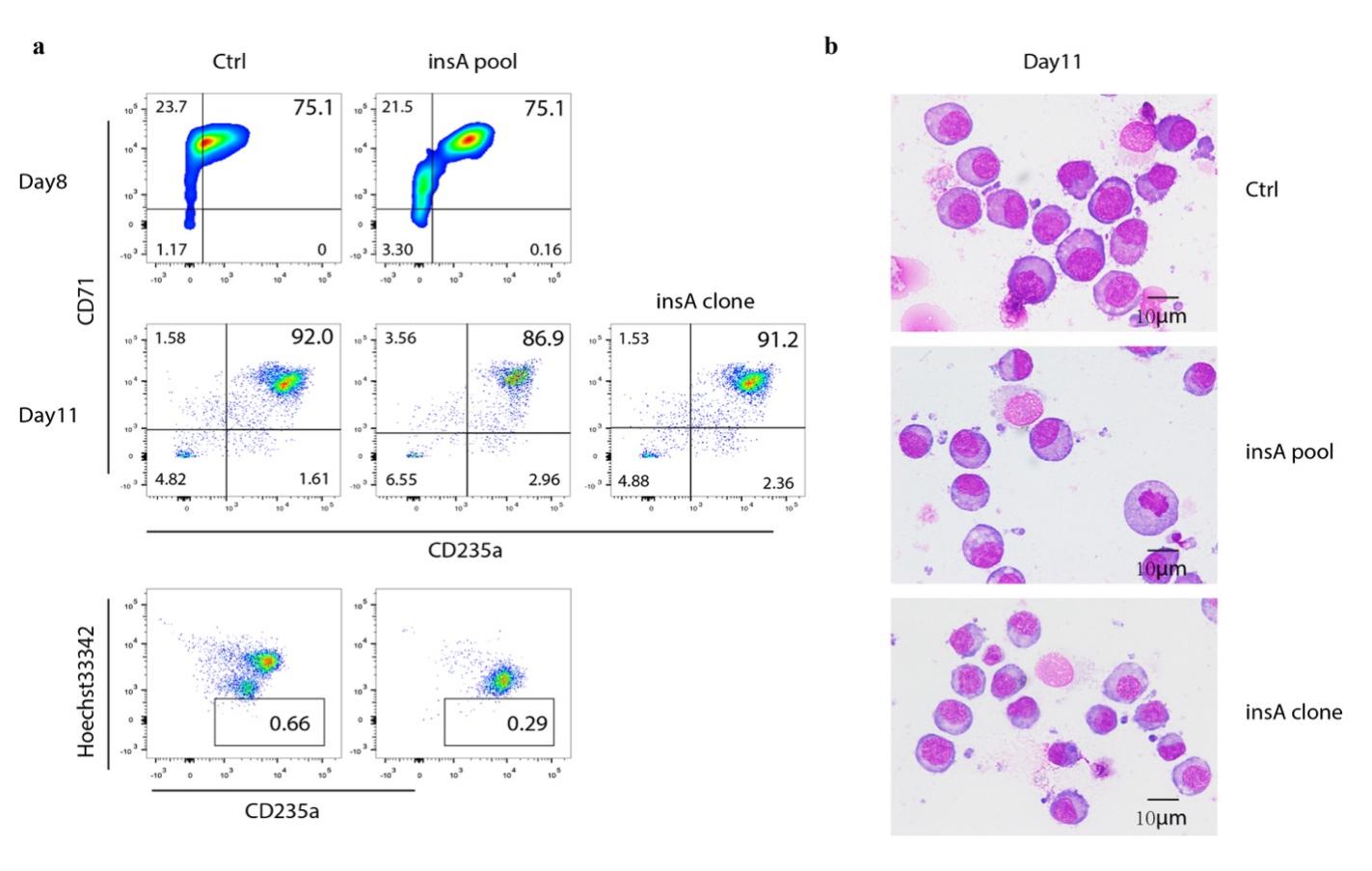
Supplementary Figure 6. The impact of insA on erythroid differentiation in HUDEP-2 cells.** a. Flow cytometry analysis of the surface markers of CD235a and CD71 (top) and enucleation rate (bottom) in day 8 and 11 after differentiation in insA and control HUDEP-2 cells. b. Representative images of Giemsa staining in control and insA pool or clone HUDEP-2 cells.


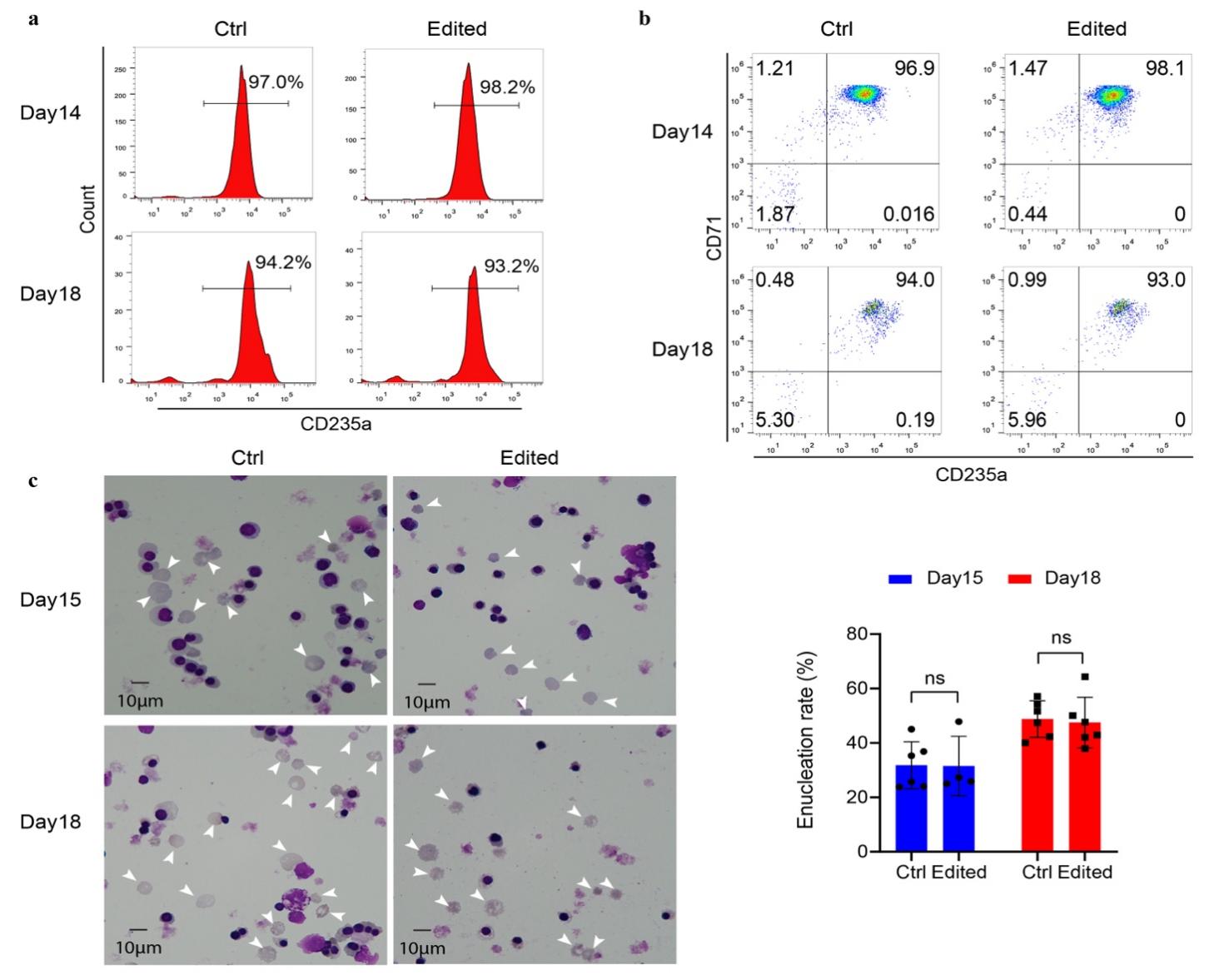


**Supplementary Figure 7. The impact of insA on erythroid differentiation in CD34^+^ HSPCs from healthy donors.** a. Flow cytometry analysis of surface marker of CD235a in edited CD34^+^ HSPCs shown as histogram. b. Flow cytometry analysis of CD235a and CD71 in edited and control cells. c. Left: Enucleation rate analysis with Giemsa staining in different time points of differentiated CD34^+^ cells (objective lens, 100×). The white arrows indicate the reticulocytes. Right: the statistical results of the enucleation rate by calculating at least four cytospins in each group.

**
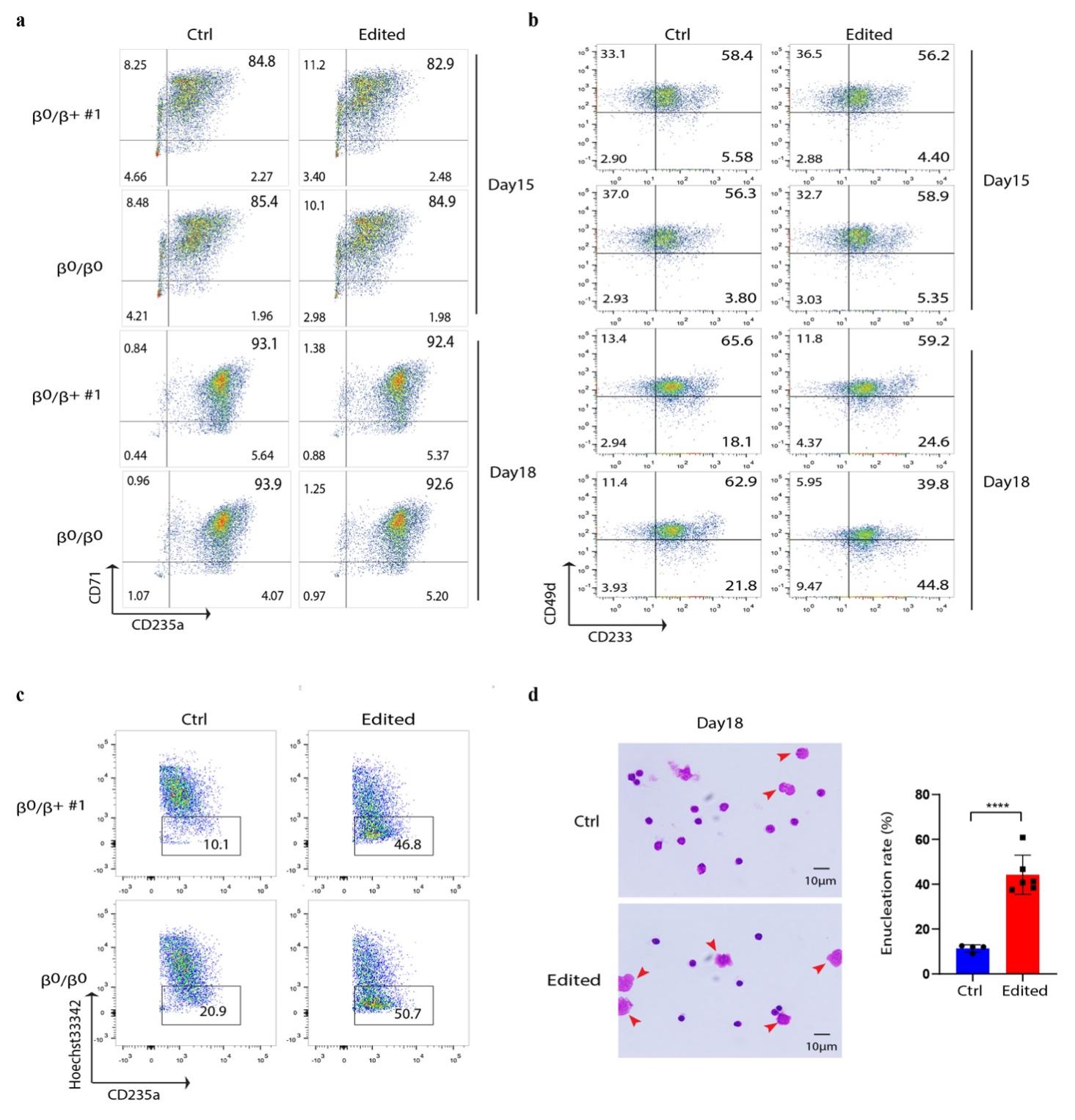
Supplementary Figure 8. The impact of insA on erythroid differentiation in CD34^+^ HSPCs from β-thalassemia patients.** a-b. Flow cytometry analysis of surface markers CD235a and CD71 (a), and CD49d and CD233 (b) at day 15 and day 18 edited and control cells. c. Hoechst 33342 and CD235a were used to monitor the enucleation rate of control and edited cells. d. Left: representative images of Giemsa staining at day 18 differentiated CD34^+^ cells (objective lens, 100×). The red arrows indicate the reticulocytes. Right: the statistical results of the enucleation rate by calculating at least four cytospins in each group.


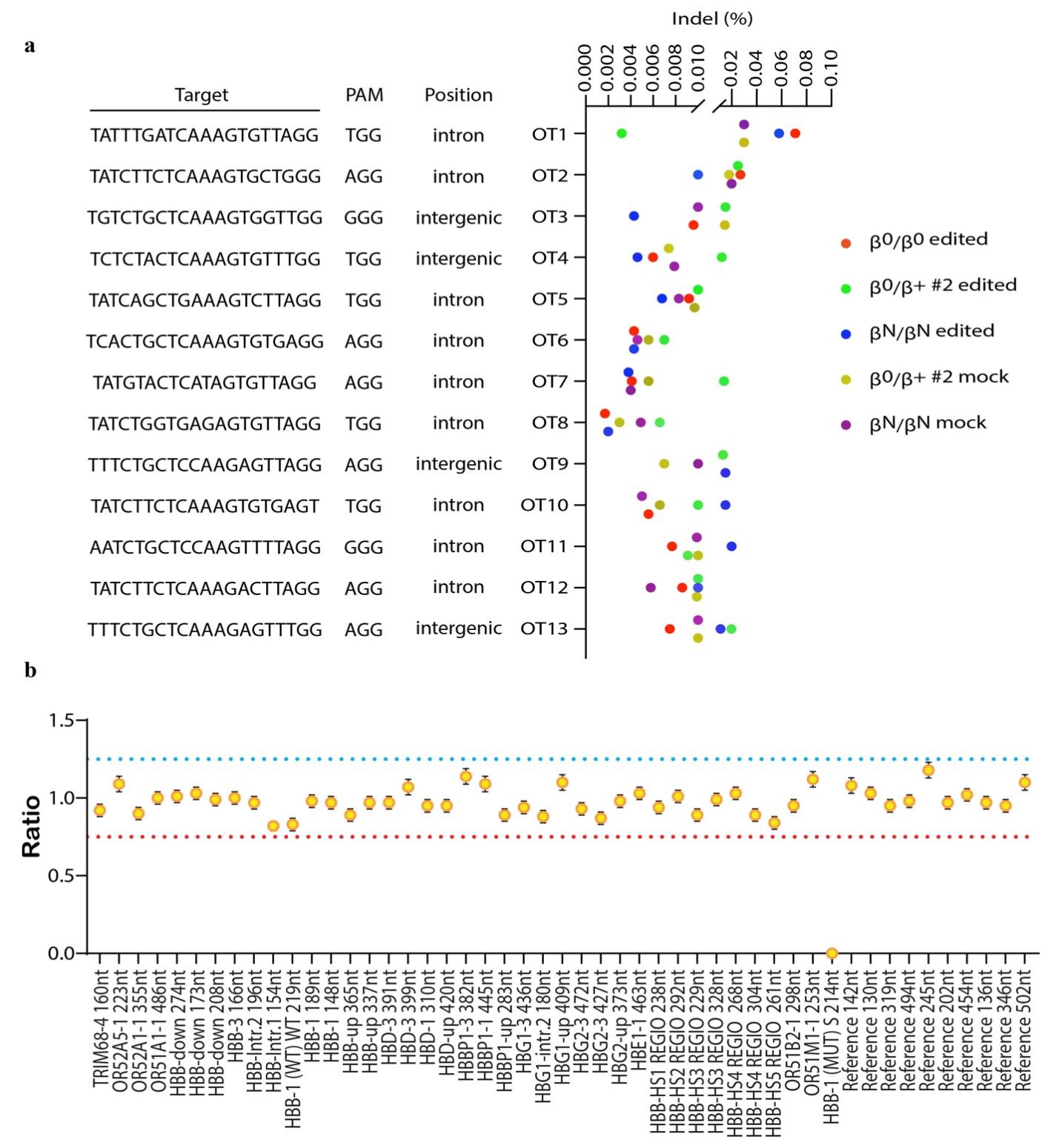


**Supplementary Figure 9. Introducing insA had no significant off target effect.** a. Deep sequencing analysis of top 13 potential off-target sites predicted by Cas-OFFinder within edited human CD34^+^ HSPCs. b. Multiplex ligation-dependent probe amplification (MLPA) analysis of the intact of the whole β-globin cluster in edited CD34^+^ HSPCs. The ratios of all probes ranged from 0.75 (red dot line) to 1.25 (blue dot line), which indicates normal copy number.

**
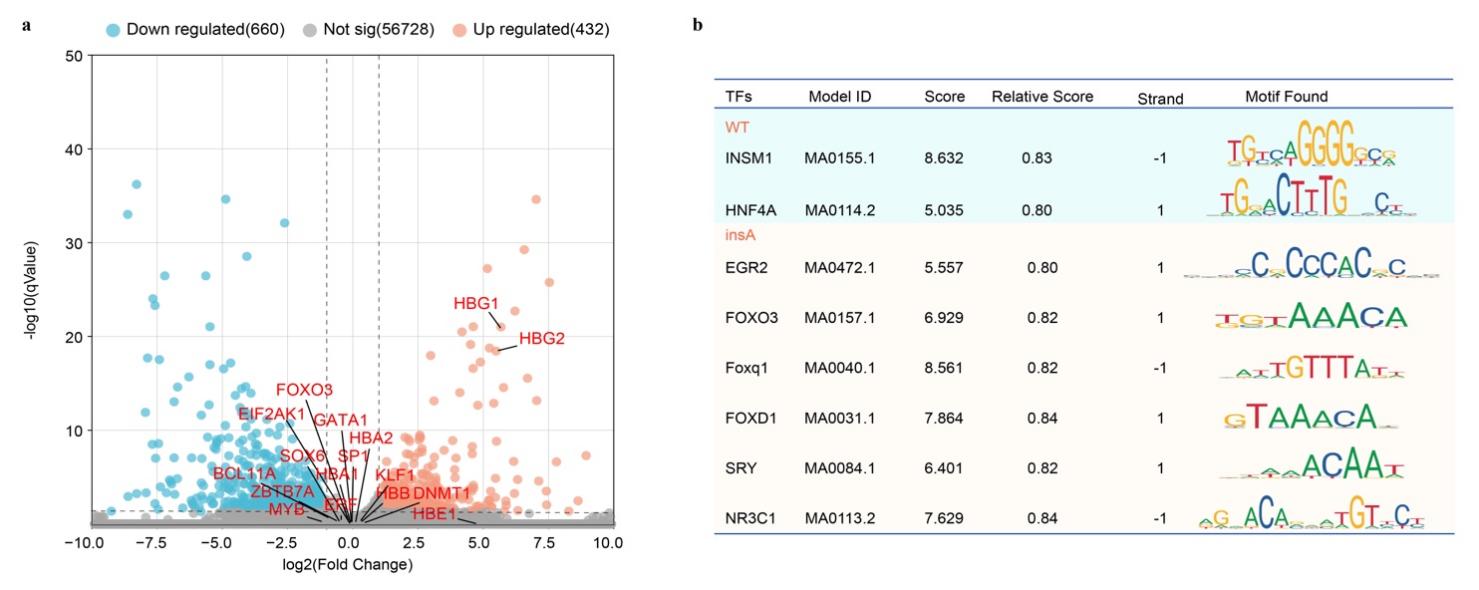
Supplementary Figure 10. InsA mutation creates a *de novel* site for activator FOXO3.** a. RNA-seq was performed in 3 insA and control HUDEP-2 clones respectively. The orange and blue circles denote significant genes according to the screening standards: |fold change (FC)| >2, qValue < 0.05. b. The predicted transcription factors and their binding motifs in the indicated position before (WT) and after introducing insA by JASPAR.

**
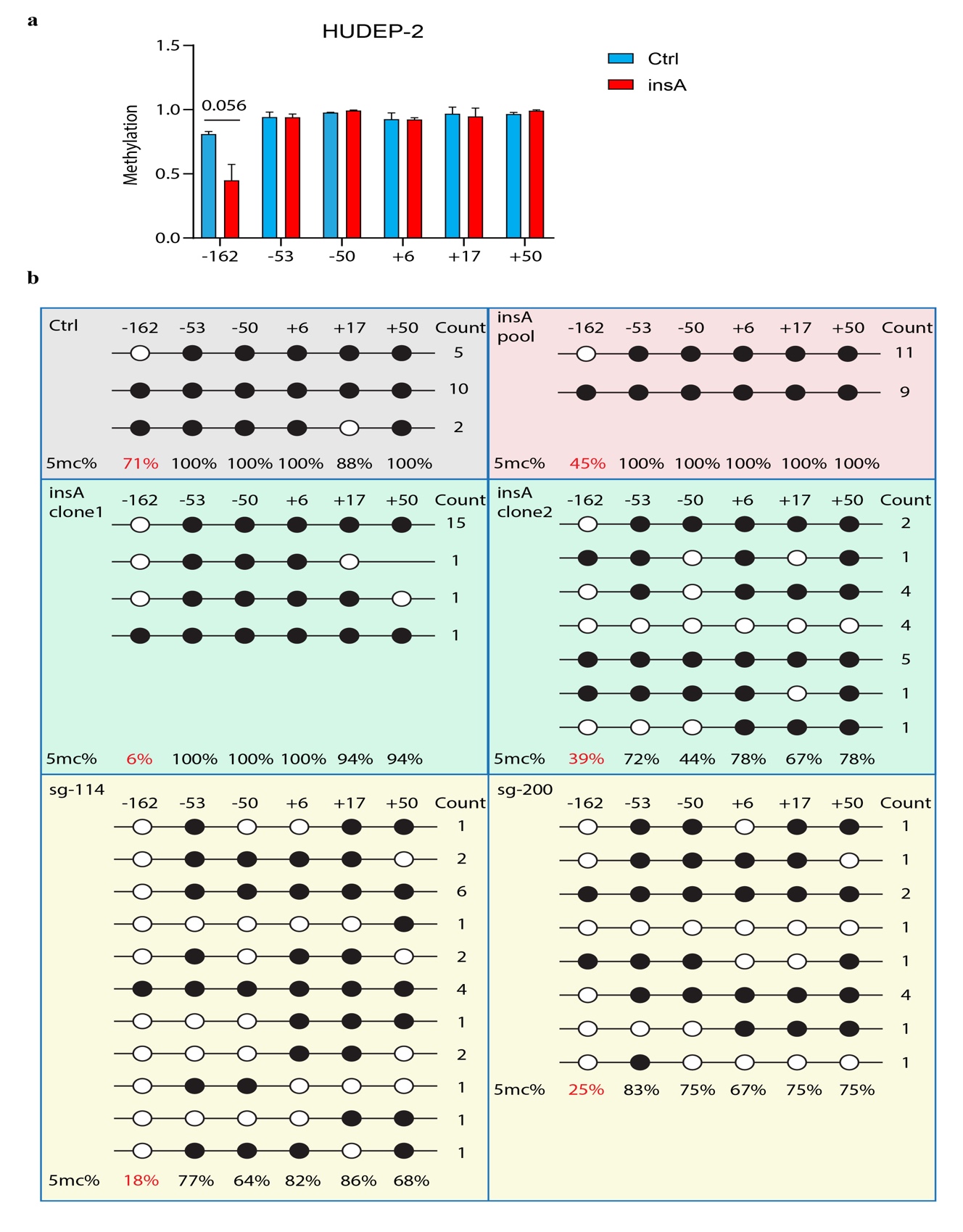
Supplementary Figure 11. insA demethylated -162 site on *HBG* promoter in HUDEP-2 cells.** a-b. Analysis of methylation levels at CpG sites (indicated by the distance relative to the transcription start site, TSS) in the *HBG* promoter, evaluated by sequencing (a) and clone sequencing (b). Data were generated with wild type, insA clone and sg-114, sg-200 HUDEP-2 cells. Each row of six CpG sites within a group represents a single bisulfite-treated clone with methylated CpGs (●) or unmethylated CpGs (○).

**
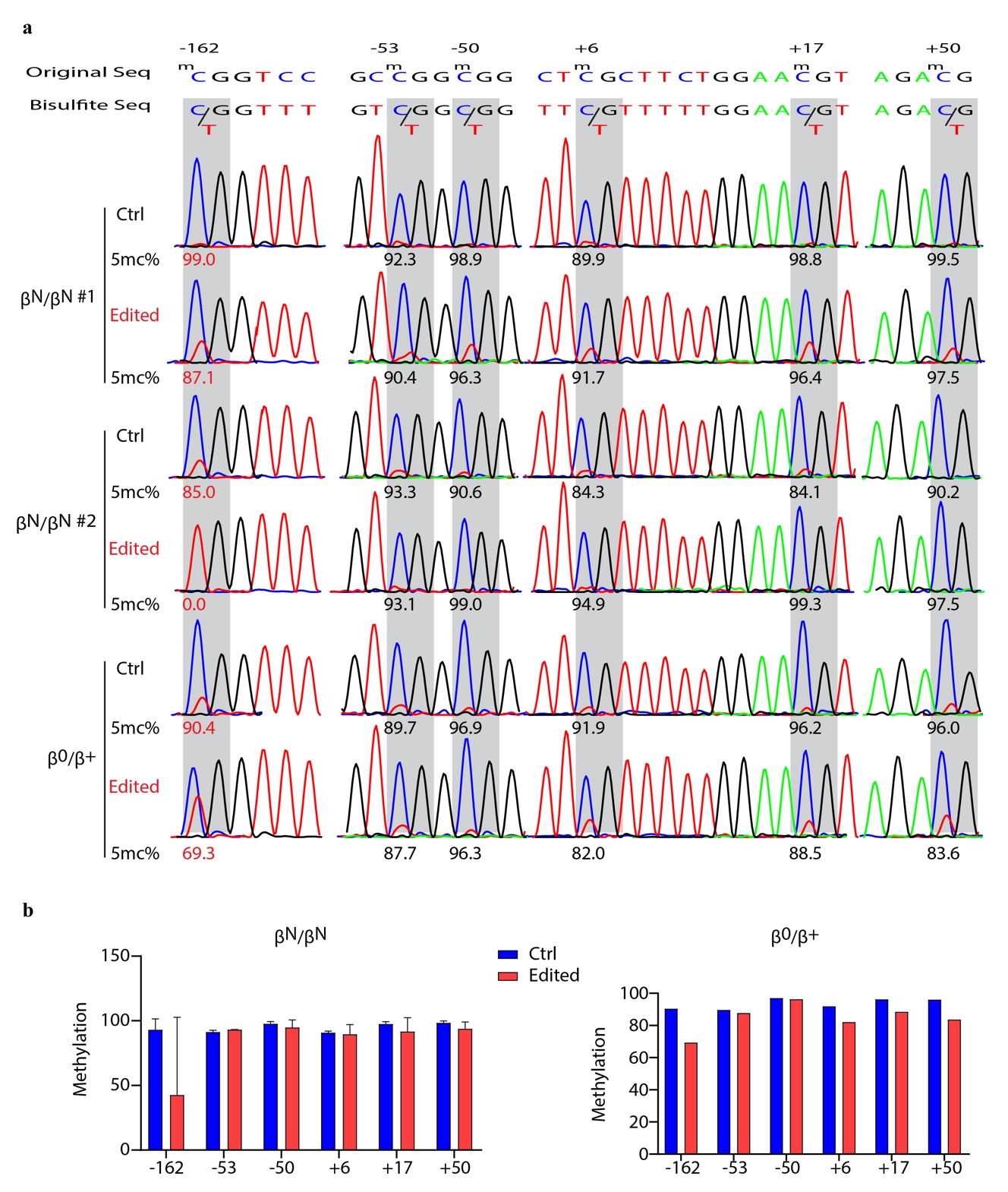
Supplementary Figure 12. insA demethylated -162 site on *HBG* promoter in CD34^+^ HSPCs.** a. Methylation levels of the *HBG* promoter were determined by the BS-seq method for the edited and unedited CD34^+^ HSPCs. Bisulfite-treated DNA was used to perform PCR. Sanger sequencing was performed to detect the methylation levels. Because an unmethylated C is represents a methylated C allele (highlighted in grey) according to the BS-seq method. The methylation levels of each CpG dinucleotide (C/(C+T)%) are indicated under the chromatograph. b. Statistical results of methylation levels detected by BS-seq method in CD34^+^ HSPCs from healthy (left panel) and β-thalassemia patient (β^0^/β^+ #1^, right panel).

**Supplementary Table 1. Primers and probes used in this study.**

| Purpose | Gene/loci | 5’ primer/WT probe  Primer/probe sequence (5’-3’) | 3’ primer/MT probe  Primer/probe sequence (5’-3’) |
| --- | --- | --- | --- |
| Real-time qPCR | ***HBG*** | GGTCATTTCACAGAGGAGGACAAG | CCAAAGCTGTCAAAGAACCTCTG |
|  | ***HBB*** | GCACGTGGATCCTGAGAACT | CACTGGTGGGGTGAATTCTT |
|  | ***HBA*** | TGCCGACAAGACCAACGTCA | GTGGGGAAGGACAGGAACAT |
|  | **β-actin** | GGGAAATCGTGCGTGACATT | GGAGTTGAAGGTAGTTTCGTG |
| ChIP-qPCR | ***HBG* (-162)** | AAGCAGCAGTATCCTCTTGGG | TCAAGGCTATTGGTCAAGGCA |
|  | ***HBG* distal** | tgcaatatgtgcagctttgttg | gagagctctaccttcccctaac |
|  | ***HBG* proximal** | agagtatccagtgaggccagg | TAGCCTTGTCCTCCTCTGTGA |
|  | ***GAPDH*** | GCTCTCTGCTCCTCCTGTTC | CCGTTGACTCCGACCTTCAC |
|  | ***MYOD*** | CCCAACTGTGTGATTTTGTGGA | CAGACTGTCATCCCCACCACA |
|  | ***FASL*** | cgatagcaccactgcactcc | agatcagaggctgcaaaccag |
|  | ***HBB*** | AAGTCAGGGCAGAGCCATCTA | GGCCTCACCACCAACTTCATC |
| Dual luciferase | ***HBG* MT promoter** | CACCCATCCTTAGATTGAGAGAAGTC | GGCGTCTGGACTAGGAGCTTATT |
|  | ***HBG* insA promoter** | GTCTTTTAGCCGCCTAAACACTTTGAGCAG | CTGCTCAAAGTGTTTAGGCGGCTAAAAGAC |
|  | ***FOXO3* CDS** | AAGATGGCAGAGGCACCGG | GATCCTTCAGCCTGGCACC |
| EMSA | **-1368/-1369** | TTTTAGCCGCCTAACACTTTGAGCA | TTTTAGCCGCCTAAACACTTTGAGCA |
| Bisulfite sequencing | ***HBG* 1st** | TTAAAAATTTTGGATTTATGTTTA | CAAATTACCAAAACTATCAAAAAACC |
|  | ***HBG* 2nd** | TTAAATTATAGGTTTTATTGGAGTT | AATCAAAAAATACCACAAATCC |
| Edited | ***HBG* insA sgRNA** | GATCTGCTCAAAGTGTTAGG | CCTAACACTTTGAGCAGATC |
|  | **AAVS1 sgRNA** | GTCACCAATCCTGTCCCTAG | CTAGGGACAGGATTGGTGAC |
|  | ***HBG* for edited validation** | tcacccatccttagattgagagaag | gtatgtatgtaggcacccgatg |
|  | **insA KO sgRNA** | ATCTGCTCAAAGTGTTTAGG |  |
|  | **insA KO donor** | CAGCTTTGTTGCGCAGGTCAACATGTATCTTTCTGGTCTTTTAGCCGCCTAACACTTTGAGCAGATATAAGCCTTACACAGGATTATGAAGTCTGAAAGG | |
| Off-target | ***OT1*** | ACATGCCCAAGACCACACAA | GCCTGAAAGGGTTTTGACTCTG |
|  | ***OT2*** | CTGCTGGTTTCAGGAGATCCTC | GAATGAATTGAGAGACAGGCAGC |
|  | ***OT3*** | ATGAATCCGAGAGAGGCGAG | GGTCGTGAACTTCAGCCACTA |
|  | ***OT4*** | AGTTGCCAAGCCTGTACCTAAA | GGACCTCTTGAGAAGGGAGGT |
|  | ***OT5*** | TATAGCCACACAGGGAAGGCT | AGGTAGAGTGGGGCTGATCAC |
|  | ***OT6*** | AGACCAATATGTTTGCCAGGAACT | AGTGCCTCCCAGGGTTTTATTC |
|  | ***OT7*** | TGCCAGAATGTGCTTCCATGAA | TTCCAATGCTGATGGAGAAAGGG |
|  | ***OT8*** | TTCCAATGCTGATGGAGAAAGGG | CTGGAACGCTTTCTCCCGA |
|  | ***OT9*** | TCTTGCCAGCCTTGGAACTT | CCCAGGCCACACTTCTTCAA |
|  | ***OT10*** | AGCATGTCGGGTAAGACCAG | GTGACTGGGATGAGGACAGG |
|  | ***OT11*** | ATTCCCTGGTAAGGGCTGGA | TTCAGACAGGCAACGTGCAG |
|  | ***OT12*** | TTTACCAAAACTGGGTGAGCG | TGAGTTGGGAGACAGATGACAAA |
|  | ***OT13*** | CCAGAGTGAGCAGATATCACAC | GGTGTGAAATAATGGTGGAAGGA |
| Multiplex editing | ***HBG-114*** | gtttgccttgtcaaggctat | ATAGCCTTGACAAGGCAAAC |
|  | ***HBG-117*** | cttgtcaaggctattggtca | TGACCAATAGCCTTGACAAG |
|  | ***HBG-200*** | cattgagatagtgtggggaa | TTCCCCACACTATCTCAATG |
| Intergenic region deletion detection | ***In-test (purple)*** | GTGTTTTAGGCATAGGTCCAGGATT | CTATTCCTGCACTGAAACTGTTGC |
|  | ***Ex-ctrl (green)*** | GCCATATCTGAGAGTCTGGTTGG | GAGTTGCAGTGAGCCGAGATCAT |

**Supplementary Table 2. *HBB* genotype of β-thalassemia patients.**

| Patients | Genotype |
| --- | --- |
| β^0^/β^0^ | *HBB*: c.126_129delCTTT/c.92+1G>T |
| β^0^/β^+^ ^#1^ | *HBB*: c.126_129delCTTT/c.316-197C>T |
| β^0^/β^+^ ^#2^ | *HBB*: c.113G>A/c.316-197C>T |
| β^+^/β^+^ | *HBB:* c.-78A>G/c.316-197C>T |

**Supplementary Table 3. Primary xenotransplant of cas9 RNP edited CD34^+^ HSPCs to NCG-Kit-V831M mice.**

| Donor | Cell number for each mice | Cas9 | sgRNA |
| --- | --- | --- | --- |
| β^0^/β^0^ | 1.67E+05 | 100 μg | 2000 pmol |
| β^0^/β^+^ ^#1^ | 3.00E+05 | 100 μg | 2000 pmol |
| β^0^/β^+^ ^#2^ | 6.40E+05 | 100 μg | 2000 pmol |
